# Combined LCA and Green Metrics Approach for the Sustainability Assessment of an Organic Dye Synthesis on Lab Scale

**DOI:** 10.3389/fchem.2020.00214

**Published:** 2020-03-31

**Authors:** Maria Laura Parisi, Alessio Dessì, Lorenzo Zani, Simone Maranghi, Sanaz Mohammadpourasl, Massimo Calamante, Alessandro Mordini, Riccardo Basosi, Gianna Reginato, Adalgisa Sinicropi

**Affiliations:** ^1^R^2^ES Lab, Department of Biotechnology, Chemistry and Pharmacy, University of Siena, Siena, Italy; ^2^Center for Colloid and Surface Science-CSGI, Florence, Italy; ^3^National Research Council, Institute for the Chemistry of OrganoMetallic Compounds (CNR-ICCOM), Florence, Italy; ^4^Department of Chemistry “U. Schiff”, University of Florence, Sesto Fiorentino, Italy

**Keywords:** life cycle assessment, green metrics, sustainability assessment, organic dyes, solar cells, lab scale, synthesis

## Abstract

New generation photovoltaic devices have attracted much attention in the last decades since they can be efficiently manufactured employing abundant raw materials and with less-energy intensive processes. In this context, the use of powerful environmental assessment is pivotal to support the fine-tuning of solar cells fabrication and hit the target of manufacturing effective sustainable technological devices. In this work, a mass-based green metrics and life cycle assessment combined approach is applied to analyze the environmental performances of an innovative synthetic protocol for the preparation of organic dye **TTZ5**, which has been successfully proposed as sensitizer for manufacturing dye sensitized solar cells. The new synthetic strategy, which is based on the C-H activation process, has been compared with the previously reported synthesis employing classic Suzuki-Miyaura cross-coupling chemistry. Results highlight the contribution of direct energy consumption and purification operations in organic syntheses at lab scale. Furthermore, they demonstrate the usefulness of the environmental multifaceted analytic tool and the power of life cycle assessment to overcome the intrinsic less comprehensive nature of green metrics for the evaluation of organic synthetic protocols.

## Introduction

In order to match the target of the EU Renewable Energy Directive (EU, 2018) and the Circular Economy Action Plan (EC COM, 2019; 190 final, 2019), the research and development activity on photovoltaics (PV) should cope in looking for more eco-friendly manufacturing solutions. Since PV devices are known to be an emission-free technology during their operative phase, the major efforts in terms of sustainability should result in minimizing the environmental impact associated with the production of the cell and module components and the end-of-life phase (Bravi et al., 2010).

This matter is of paramount importance especially for innovative PV, and particularly for the so-called last generation technologies, that stand out for the use of materials alternative to traditional semiconductors. In recent years, relevant results have been obtained in this field in terms of efficiency and technological advancement (A.T. Kearney Energy Transition Institute, 2017), thanks to a lively and very productive research activity developed at lab scale (Polman et al., 2016; Luceño-Sánchez et al., 2019). In this context, the adoption of an eco-design approach becomes essential to pursue the improvement of the eco-profile of products (Maranghi et al., 2019; Parisi et al., 2020) and the environmental sustainability assessment offers a powerful tool to achieve this goal. The panorama of environmental sustainability assessment methods offers a wide range of possibilities (Sheldon, 2017, 2018). Above all, a multivariate approach combining mass-based green metrics and life cycle assessment (LCA) affords an extremely useful tool for evaluating the environmental impact of processes, as already demonstrated in the bulk and fine chemicals production, as well as in pharmaceutical and nanotechnology sectors (Eckelman et al., 2008; Gałuszka et al., 2012; Sheldon, 2012, 2016; Barberio et al., 2014; Roschangar et al., 2015).

The concurrent use of these different tools allows to combine the versatility and practicality of green metrics with the detailed environmental screening that can be obtained through the implementation of an all-encompassing assessment like LCA. Indeed, in such a way it is possible to take in consideration relevant information such as resource efficiency and energy requirements together with all of the possible hotspots associated to the investigated systems.

Although this combined approach is quite often implemented at the industrial R&D level (Cespi et al., 2020), its application for product and processes eco-design at lab scale is not so diffused (Pini et al., 2020). Indeed, notwithstanding the increasing and recognized importance of Life Cycle Thinking (LCT) approaches also for laboratory scale procedures (Allen et al., 2010), the application of the LCA methodology requires the compiling of a comprehensive inventory of data that, when appropriate information can be found, is always a laborious and very time-demanding procedure.

So far, examples of such combined sustainability assessment are absent in the scientific literature concerning the synthesis of innovative materials for PV. For instance, in a recent work by Grisorio et al. (2015), a green metrics analysis has been applied for the evaluation of the environmental sustainability of an innovative chemical process used to prepare a solar cell component, but not in the perspective of a LCT approach.

**Figure 1:**
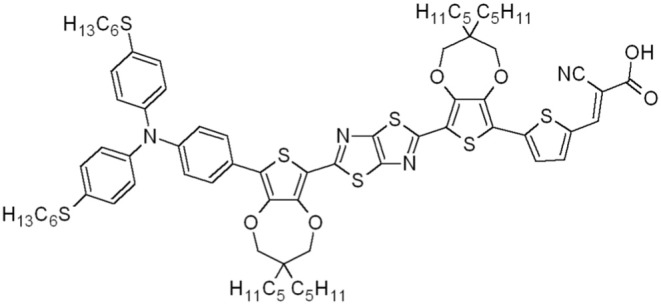
Structure of dye **TTZ5**.

In this work, a mass-based green metrics and LCA combined approach is applied to obtain a gate-to-gate assessment on two new alternative synthetic protocols, specifically designed to scale-up the production of the organic dye **TTZ5** (Figure 1), and compare their environmental performances with those of its original preparation (Dessì et al., 2014). Compound **TTZ5**, bearing a thiazolo[5,4-*d*]thiazole (TzTz) ring as its central unit, has been successfully employed as a sensitizer for the preparation of photocatalysts for hydrogen production (Dessì et al., 2018) and the manufacturing of dye sensitized solar cells (DSSCs) (Dessì et al., 2014, 2015). This innovative photovoltaic technology dates back to 1991 and was proposed as a more versatile and cheaper alternative to silicon based photovoltaic (PV) devices. Indeed, DSSCs proved to be more competitive in terms of ease of production, reduction in the use of hazardous substances, manufacturing costs and raw materials availability compared with other photovoltaic devices (Hagfeldt et al., 2010; Gong et al., 2017). However, the efficiency of these devices is strongly dependent on the sensitizer (Parisi et al., 2013, 2014). From this perspective, dye **TTZ5**, when used in thin film DSSCs (photoanode thickness ~5.5 μm) drew great attention (Dessì et al., 2014) showing efficiencies up to 7.71%, which are superior to the Ru-based sensitizer **Z907** (η 5.51%) measured in the same conditions, and approaching the 11.9% record efficiencies registered for champion DSSCs (NREL, 2019). For these reasons, **TTZ5** is particularly suitable for application in transparent and opaque thin-layer cells (Dessì et al., 2014), which are a common choice for the application in Building Integrated Photovoltaic (BIPV) systems.

## Materials and Methods

### Synthetic Approach

**Scheme 1:**
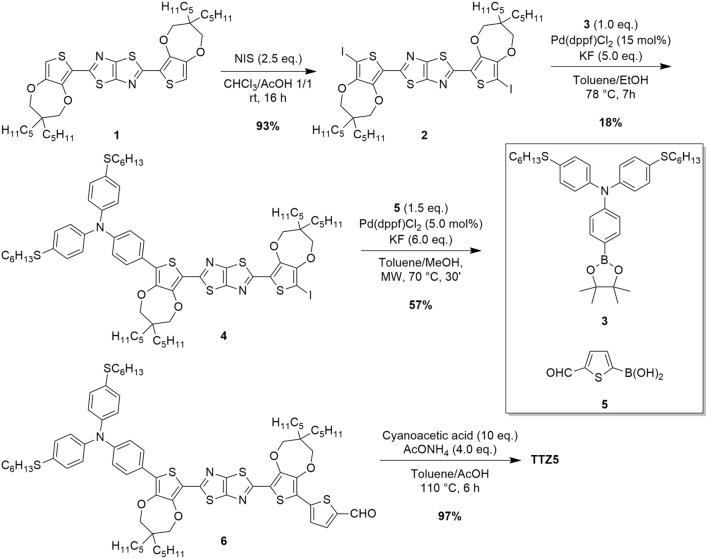
Functionalization of TzTz **1** with donor and acceptor groups through Suzuki-Miyaura cross-couplings.

**Scheme 2:**
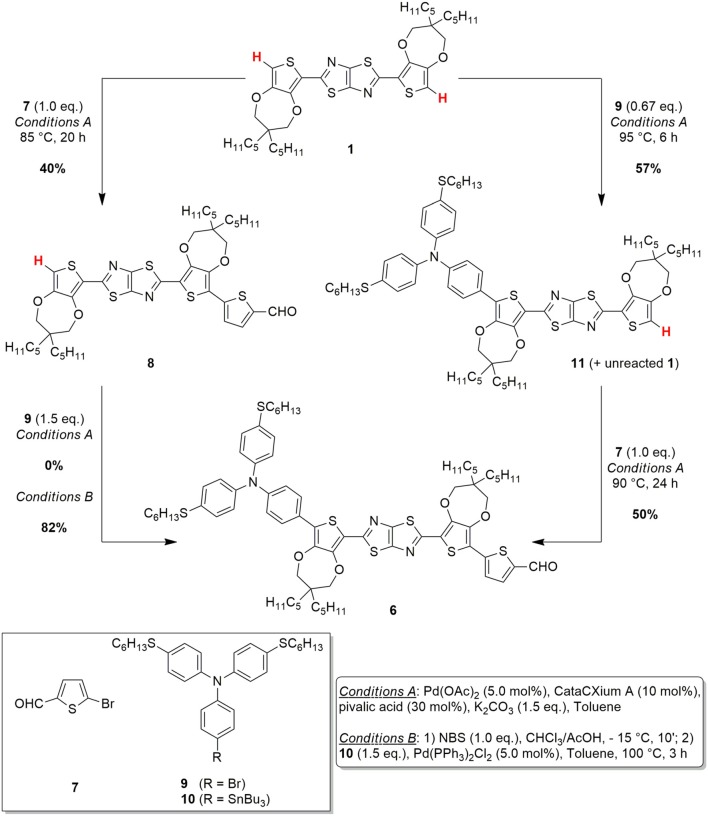
C-H activation/Stille-Migita route (on the left) and C-H activation route (on the right) for the conversion of the starting material **1** to the advanced intermediate **6**.

In the last years, **TTZ5** has found application as a photosensitizer in the photocatalytic hydrogen production (Dessì et al., 2018) and in transparent thin-layer DSSCs (Dessì et al., 2014), which are very attractive for the production of colored, transparent modules to be applied in building-integrated photovoltaics. However, fulfilling this aim would require the preparation of large-scale panels, in turn demanding the development of a reliable and sustainable gram-scale preparation of the dye. The original synthesis of **TTZ5** required three main synthetic operations to obtain the key intermediate aldehyde **6**: (a) the preparation of thiazolothiazole-based spacer **1** (Scheme 1), (b) the preparation of boronic ester **3**, containing the donor group (Scheme 2) and (c) the introduction of the donor and the acceptor moieties through two sequential Suzuki-Miyaura cross-couplings (Scheme 1). Finally, the anchoring group cyanoacrylic acid was introduced following the well-established Knoevenagel condensation protocol on aldehyde **6** (Dessì et al., 2014). The undeniable Achilles' heel of the old synthetic approach, which hampered the scale-up of **TTZ5**, was the desymmetrization of thiazolothiazole-based spacer **1**: following a straightforward double electrophilic iodination, the Suzuki-Miyaura coupling of diiodide **2** with boronic ester **3** proved troublesome, since the reaction had to be stopped before the complete conversion of the starting material **2**, in order to minimize the second coupling with the residual carbon-iodine bond in compound **4**. Moreover, undesired dehalogenation of both the starting material **2** and the product **4** decreased the yield, making the chromatographic purification difficult. As a result, only a small amount of the final compound could be obtained in pure form.

In order to design a more sustainable and scalable synthetic procedure of dye **TTZ5**, we decided to change our approach to the functionalization of building block **1**. Our choice was to move from a classic Suzuki-Miyaura cross-coupling to the exploitation of a direct arylation protocol. In this case, in fact, the formation of a new carbon-carbon bond could be performed avoiding the use of any preformed organometallic reagent and reducing the number of the synthetic steps (Zani et al., 2019). Inspired by Liu's work about the C-H functionalization of a central heteroaromatic core with different electron-rich and electron-poor bromides (Lu et al., 2017), we initially explored the single direct arylation of starting material **1** with the electron-poor bromide **7** (Scheme 2). Application of Liu's reaction conditions, which consisted in using an excess amount of the starting material, Pd(OAc)_2_ as pre-catalyst, the bulky electron-donating phosphine CataCXium® A as ligand, pivalic acid as the acid additive, inexpensive K_2_CO_3_ as the base and *N*,*N*-DMF as the solvent at 110°C, allowed to isolate pure intermediate **8** with 28% yield.

Unfortunately, part of the starting material **1** decomposed under these conditions and just a small fraction of its excess could be recovered at the end of the reaction. Despite that, the simple change of *N*,*N*-DMF with a less polar solvent such as toluene and a reduction of the reaction temperature to 85°C allowed to minimize undesired side-reaction and to employ just a stoichiometric amount of thiazolothiazole **1**. Under these conditions, a 70% conversion of the starting material **1** could be achieved, leading to a 40% yield of the desired aldehyde **8** as a pure product. Disappointingly, when the same conditions were tested to introduce the donor group by reaction with bromide **9**, no conversion to product **6** was observed. To achieve this transformation, a quantitative electrophilic bromination of intermediate **8** and a subsequent Stille-Migita cross-coupling with stannane **10** proved necessary, affording the desired product in 82% yield. Accordingly, following this C-H activation/Stille-Migita cross-coupling route, the desired advanced intermediate **6** could be isolated in a two steps sequence, with an overall yield of 33%, leading to a good improvement in terms of yield and selectivity compared to the original synthetic strategy. In addition, application of this route allowed to scale-up the preparation of compound **6** to a quantity of ~1.3 g, much higher than that obtained with the original synthetic sequence.

However, the sustainability of the whole process appeared still limited by the employment of the tin-containing reagent (**10**) and the use of quite harsh conditions: clearly the possibility to perform two subsequent C-H activation processes would be largely preferred. To reach this goal, we decided to explore a different approach, inspired by those previously reported for the direct arylation-based synthesis of D-π-A dyes for DSSCs (Lin et al., 2015; Lu et al., 2017), and consisting in the reversal of the order in the introduction of the donor and acceptor groups (Scheme 2). Thus, the direct arylation of the starting material **1** was first performed with donor bromide **9** and then the resulting intermediate **11** was reacted with electron-poor bromide **7**.

The first step of the synthesis was indeed accomplished using the same reaction conditions mentioned above, with a slight increase of the temperature and using an excess of the starting material **1**. The reaction proceeded smoothly, with a complete conversion of donor bromide **9**; unfortunately, however, a complete chromatographic separation of product **11** and unreacted starting material **1** was almost impossible, hampering the isolation of pure **11**. Despite that, such 1:1 mixture of **11** and **1** could be successfully reacted with electron-poor bromide **7** to give, under the usual conditions, pure compound **6**, which was isolated after chromatographic purification. Unfortunately, the excess of reagent **1** used in the first step could not be recovered at the end of the second step, since it underwent a double direct arylation with bromide **7**. The overall yield of this C-H activation route was 29%, comparable to the previous C-H activation/Stille-Migita route in terms of yield, although a larger amount of starting material **1** was consumed.

**Scheme 3:**
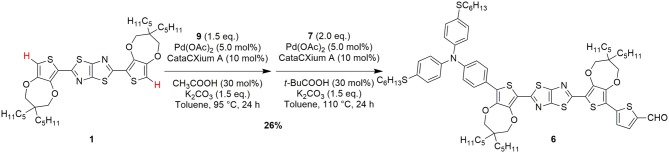
One-pot C-H activation route for the conversion of the starting material **1** to the advanced intermediate **6**.

Finally, to overcome the problem of purification of intermediate **11** and trying to minimize the presence of unreacted **1**, we decided to test a *one-pot* direct arylation protocol (Scheme 3), aiming to obtain compound **6** directly from the starting material **1** and to avoid the isolation of any intermediate. To achieve this goal, an optimization of the reaction conditions was carried out using different Pd catalyst and conditions. Thus, using tri-*tert*-butylphosphine as a ligand no conversion of the starting material **1** was obtained, while using tricyclohexylphosphine or CataCXium® A provided the desired product **6** with yields of 13% and 19%, respectively. Finally, we found that, for the introduction of the donor group, acetic acid was superior to pivalic acid, since it reduced the relevance of possible side-reactions such as a partial oligomerization of **1**.

Following this optimized *one-pot* C-H activation route, compound **6** was thus obtained in 26% yield after purification (isolated amount: 0.24 g). Clearly, from a practical point of view, despite the overall yield of this process was a bit lower than the previous ones, the possibility of accessing intermediate **6** in a single synthetic step, without using stoichiometric organometallic reagents and avoiding expensive and time-consuming purification procedures, makes this route more intriguing especially for large scale application (see Table 1).

**Table 1:** Comparison of the three alternative routes for the preparation of **TTZ5** in terms of yield, number of steps and required chromatographic purifications.

**Route**	**Yield (%)^**a**^**	**N^**°**^ Steps^a^**	**N^**°**^ chromatographic purifications^a^**
Suzuki-Miyaura	9%	4 (+1)^b^	2 (+1)^b^
C-H/Stille	32%	3 (+1)^b^	2
One-pot C-H Activation	25%	2	1

a From compound **1** to final product **TTZ5**.

b *In comparison with the One-pot C-H Activation Route, one step more is necessary for the preparation of the organometallic reagents (boronic ester **3** and stannane **10** for Suzuki-Miyaura Route and C-H/Stille Route, respectively)*.

### Environmental Sustainability Assessment

**Figure 2:**
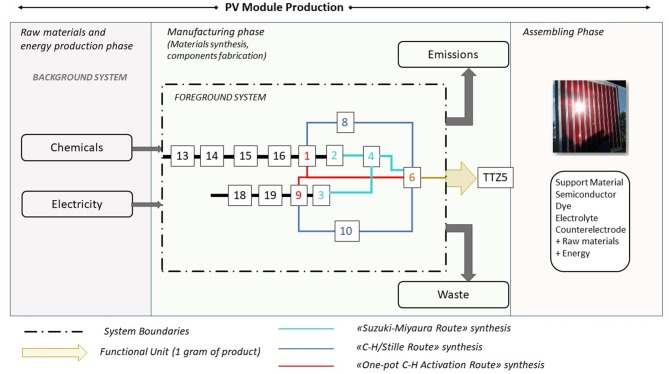
PV module production process: sketch of the synthetic routes and system boundaries of this study.

As anticipated in the introduction, an important aim of this work is the calculation of the eco-profile of the photosensitizer **TTZ5** produced via the three above-mentioned alternative synthetic routes, to assess if an environmental sustainability improvement can be obtained while looking for scalability of the manufacturing procedure. A generic PV module production process is synthetically represented in Figure 2: it starts from the acquisition of raw materials, encompassing the fabrication process of all the components and it ends with the assembling phase.

The implemented approach is a gate-to-gate analysis exclusively focused on the manufacturing phase of the DSSC photosensitizer, using both green metrics and LCA calculations. The reason of this choice arises from the necessity of evaluating exclusively the obtained improvements (if present) derived from the change of reaction strategies for the production of target molecule **TTZ5**. The foreground system includes the original synthesis and the two new alternative synthetic routes to obtain **TTZ5**. An overview of the process is sketched in the figure, where the three routes are distinguished with different colors [Compounds **13**, **14**, **15**, **16**, **18**, **19** are numbered according to the synthetic procedure described in (Dessì et al., 2014), see Schemes 1, 2].

In the following sub-sections, some methodological details are given.

#### Green Metrics

##### E-factor

The E-factor (EF) represents the quantity of waste generated (i.e., anything except the product) to produce 1 unit of the target product. Work-up substances, auxiliaries and solvents are incorporated in the definition of waste. The following equation express the value of EF:

$$\begin{array}{l} EF=\frac{\sum_{i}\;m\left({raw\;material}_{i}\right)-m_{p}}{m_{p}} \end{array}$$

Where *m*_*p*_ represents the mass of products. A higher EF means more waste and, consequently, greater negative impact. The ideal EF is zero. Lower EF have been shown to correlate well with reduced manufacturing costs which is a reflection of lower process materials input and output, reduced cost of hazardous and toxic waste disposal, improved capacity utilization and reduced energy demand. In this work, since the main purpose is a lab-scale application, EF is calculated in g/g to suitably quantify discards in laboratory synthesis.

##### Overall yield

The overall yield has always been a pillar of calculations on reaction efficiency. Considered as a “classical metrical measure” like selectivity, its relevance persists also in green metrics assessment. An implicit consideration of the overall yield is included in the EF calculation, since higher yields result in less disposal (inferior mass of byproducts, major incorporation of reactants into the target product, etc.).

##### Price estimate

In order to evaluate whether more environmentally benign syntheses provide a real financial benefit, estimation on the global price of product becomes remarkable. Calculating the cost of production of a target molecule involves incorporation of all the expenses related to the implemented raw materials. To achieve this objective, commercially available chemical prices were collected. In order to draw the attention on advantages due to waste prevention, costs related to recovered materials were subtracted to the “total waste price.” At this point, to assess the price of the product per gram, the “total waste price” is divided by the grams of obtained product. In case one of the reactants was not commercially available, but derived from a previous synthesis, its costs were incorporated into the reaction path price by adding to the “total price of waste.” This enables a “conditional cost estimate,” namely a price evaluation specific for the conditions of a synthesis (costs of reactants and their relative usage, spared materials and so on) allowing the exact appraising of financial burden on synthetic approaches and potential improvements in the perspective of a possible industrial scalability.

##### Eco-scale

Eco scale is a semiquantitative laboratory scale tool to estimate the eco-sustainability of chemical reactions based on yield, price, safety conditions, technical setup, temperature/time and ease of workup/purification. The usefulness of this tool in up-scaled processes is not significant because relative weights of the six proposed categories may differ for industrial purposes. The ideal Eco Scale value is 100, representing an “ideal reaction where a substrate A undergoes a reaction with the inexpensive reactant B at room temperature to give the product in 100% yield with a minimal risk for the operator and the environment.” For this assessment the original approach of Van Aken et al. (2006) was adopted. Thus, penalty points are assigned according to a specific ranking for each category and then subtracted by the ideal value of 100 to obtain the Eco scale rate. Eco Scale values higher than 75 are associated to an excellent performance; values between 75 and 50 are considered acceptable, while Eco Scale scores minor than 50 are assigned as inadequate.

#### LCA

According to the ISO 14040 family standards (International Organization for Standardization, 2006a,b) and the more completely elaborated ILCD Handbook Guidelines (European Commission, 2010), an attributional approach was implemented for the LCA calculation on **TTZ5** synthesis. The functional unit chosen in this study is 1 g of the target product. The same unit has been employed for the calculation of the green metrics. No allocation procedure was required since all of the environmental burdens were attributed to the final product of the process, i.e., 1 g of **TTZ5**. Data life cycle inventory (LCI) was built based on lab primary data. When needed, meta-data (conveniently customized to generate datasets for reactants, auxiliaries and solvents) and secondary data were taken from the Ecoinvent Database v. 3.4 (Wernet et al., 2016). Equipment and instruments are not included in the system modeling as capital goods, but they are accounted for in terms of energy requirement for their functioning. The life cycle impact assessment (LCIA) method employed is the ILCD 2011 Midpoint+ method (version 1.0.9, May 2016), developed by the Joint Research Centre—European Commission (European Commission, 2010) that allows to obtain single scores results expressed as Eco-Points (Pt). The Cumulative Energy Demand (CED) method (version 1.09, August 2014) is employed to quantify the use of the direct and indirect energy requirement during all the life cycle phases of the system expressing the results in units of Mega Joule (MJ) (Frischknecht et al., 2007). LCA calculations are performed with software Simapro v. 8.5.

## Results and Discussion

Table 2 shows the EF values for intermediates and **TTZ5** generation for the C-H/Stille route and the *one-pot* C-H activation route, respectively. Data concerning the Suzuki-Miyaura route are reported for comparison (more details are available in the Supporting Information).

**Table 2:** EF results for the C-H/Stille route and the one-pot C-H activation route procedures compared to the original Suzuki-Miyaura route.

**Product**	**Suzuki-Miyaura route**		**C-H/Stille route**		**One-pot C-H activation route**	
	**EF water excluded**	**EF water included**	**EF water excluded**	**EF water included**	**EF water excluded**	**EF water included**
Compound 13	27.31	29.23	27.31	29.23	27.31	29.23
Compound 14	47.92	52.09	47.92	52.09	47.92	52.09
Compound 15	562.50	600.02	562.50	600.02	562.50	600.02
Compound 16	1906.86	2000.38	1906.86	2000.38	1906.86	2000.38
Compound 1	7773.36	7971.50	7773.36	7971.50	7773.36	7971.50
Compound 2	6354.72	6513.72	–	–	–	–
Compound 8	–	–	21307.58	21740.55	–	–
Compound 18	405.79	405.79	405.79	405.79	405.79	405.79
Compound 19	1950.84	2018.41	1950.84	2018.41	1950.84	2018.41
Compound 9	1817.77	1962.55	1817.77	1962.55	1817.77	1962.55
Compound 3	5712.83	6509.90	–	–	–	–
Compound 4	49103.81	50238.93	–	–	–	–
Compound 10	–	–	1583.55	2271.85	–	–
Compound 6	94719.08	96737.07	19080.93	20240.26	28873.89	29630.56
TTZ5	93815.76	96384.21	20654.34	26505.92	28924.85	30250.26

The reported data clearly disclose the general trend of EF growing with increasing reactant complexity, and the contribution of water in its calculation. The three routes have in common five steps for the synthesis of thiazolothiazole **1** (from **13** to **1**, see Scheme 1), three steps for the preparation of the brominated triarylamine **9** (from **18** to **9**, see Scheme 2) and the last one for the synthesis of the final compound **TTZ5**, so they can be excluded from the direct comparison (gray background in Table 2). It is evident from the simple comparison of the final **TTZ5**-EF values that both the new C-H activation-based routes generated a much lower amount of waste than the original Suzuki-Miyaura protocol, with a three-to-four times decrease of the EF values. This EF-drop is due both to the smaller number of synthetic steps required by the new routes compared to the old one (one step less for the C-H/Stille route and three less for the *one-pot* C-H activation route) and the higher efficiency of the desymmetrization reaction (compound **8** C-H/Stille Route – EF: 21307.58 g/g; compound **6** *one-pot* C-H Activation Route – EF: 28873.89 g/g, to be compared with compound **4** Suzuki-Miyaura Route – EF: 49103.81 g/g). Moreover, the Suzuki-Miyaura route was penalized by a high increase of the EF for the transformation of compound **4** (49103.81 g/g) into compound **6** (94719.08 g/g) too. The same transformation was either included in the desymmetrization step of the *one-pot* C-H activation route or, at least, much more efficient in the C-H/Stille route.

Indeed, considering the evolution of water-including EF outcomes, the C-H/Stille route shows increasing values from 29.23 g/g of compound **13** to 21740.55 g/g of compound **8**, this latter being the highest one. This trend becomes comprehensible considering that the more synthetic steps are required for manufacturing a specific reactant, the more previous waste is involved in the final calculations. However, EFs are not only influenced by previous waste or mass of reactants, but also yields of reactions play an important role on material efficient consumptions, since these calculations are made on gram-of-target molecule basis. Accordingly, water-excluding EFs proved to decrease for compound **9** and **10**, compared to their previous intermediate (compounds **19** and **9**, respectively), despite the necessity of more complex reactants. This trend can be understood considering the high reaction yield obtained in both processes (98% and 100% efficiency, respectively). In fact, increasing yields correlate with larger amounts of product, which, in turn, translate in lowering of waste per gram. Compound **10** exacerbates this trend since the high yield is supported also by a lower depletion of previously synthetized reactant (which correlates with the reduction of previous waste).

As shown in Table 2, for the water-including EF values, water plays a relevant role in reducing efficiency in material preparation. Indeed, the EF of the *one-pot* C-H activation route approach demonstrated to be less influenced by water considerations. Divergence in EF values among the water-included and the water-excluded outcomes amplifies with the progressively increasing complexity of the molecules. Interestingly, results underline that even processes which do not involve depletion of water, like manufacturing of compounds **1** and **8**, exhibit dissimilar EFs. The reason arises from previous steps contribution in water consumption, proving again how EF, if suitably implemented, can represent an efficient instrument in molecular analysis recording memories of previous processes. The convergence of the EF values both in water inclusion and water exclusion for product **18**, instead, becomes obvious considering that water was not depleted in the process and the target molecule was produced only through commercially available reactants.

Finally, the EF values for **TTZ5** show a direct correlation with the high values obtained for intermediate **6**, since the final step of the synthesis is substantially identical for all routes. Regarding the overall yield for manufacturing **TTZ5** starting from compound **12** (Scheme 1), values of 4.90% and 3.75% are obtained for the C-H/Stille route and for the *one-pot* C-H activation route, respectively. In general, a significant improvement is obtained if compared to the original Suzuki-Miyaura route featuring a 0.81% overall yield. Comparing the two new synthetic protocols, the *one-pot* C-H activation route exhibits an inferior yield and higher EF values, despite an inferior number of reaction steps. The best explanation of this trend arises from the lower yield of production of the aldehydic precursor **6** using the *one-pot* C-H activation route (26%), in comparison with the overall yield of the conversion of thiazolothiazole **1** to precursor **6** using the C-H/Stille route (33%).

Concerning the overall financial burden in manufacturing **TTZ5**, it should be highlighted that costs are related not only to commercially available reactants, but also to intermediates which need to be previously synthetized. Table 3 reports the calculated overall costs for all synthetic routes. Details concerning the estimated cost of production of each intermediate are reported in the Supporting Information.

**Table 3:** TTZ5 price estimate results for the C-H/Stille route and the one-pot C-H activation route procedures compared to the original Suzuki-Miyaura route.

**Product**	**Suzuki-Miyaura route**		**C-H/Stille route**		**One-pot C-H activation route**	
	**Synthetic step total cost (€)**	**Compound cost (€/g)**	**Synthetic step total cost (€)**	**Compound cost (€/g)**	**Synthetic step total cost (€)**	**Compound cost (€/g)**
**TTZ5**	2413.52	3497.85	586.58	850.11	771.70	1118.41

The higher efficiency of the two new procedures resulted in a lower cost of the final product. Indeed, the cost of 3497.85 €/g calculated for the original process has been largely reduced to 850.11 and 1118.41 €/g for the C-H/Stille route and the one-pot C-H activation route, respectively. Again, this is related to the strong improvement of the Pd-catalyzed processes. Indeed, the most expensive steps in the previously reported synthesis were both the desymmetrization of diiodide **2** (cost of compound **4** – 1729.07 €/g) and the introduction of the acceptor moiety (cost of compound **6** – 3496.62 €/g), while desymmetrization of compound **1** was the most expensive step for both the C-H/Stille route and one-pot C-H activation route (800.70 and 1082.19 €/g, respectively). Furthermore, among all the reagents used to introduce the donor group, boronic ester **3** was the most expensive one (cost of compound **3**, 281.91 €/g vs. 88.03 €/g for compound **9** and 75.25 €/g for compound **10**).

Comparing the two optimized procedures, avoiding the use of reagent **10** and the preparation of compound **8** should have contributed, in the *one-pot* C-H activation route, to decrease the overall price of **TTZ5**. However, synthesis of the aldehydic precursor **6** in such route involves significant expenses due to the use of chromatographic purifications (costs for silica cartridge, petroleum ether and toluene amount to 78.92 €/g with the solvent contributing the most). Conversely, producing compound **6** from intermediates **10** and **8**, as in the C-H/Stille synthesis, involved major costs due to the high-price of production of these reactants, while compound **9** proved to be more economical. Starting material **1** was prepared following Scheme 1 from inexpensive reagents **13** and **14**, in turn prepared from commercially available very simple starting materials. On the other hand, compound **18** (Scheme 2), despite being obtained from commercially available chemicals, was responsible for a higher cost, mainly due to the need to isolate it through dedicated purification procedures.

Despite the general trend observed, that costs rise when more complex intermediates are used, this was not observed in the case of compound **19** and **10**. In this case, the simpler compound **19** (95.75 €/g) appears more expensive than compound **10** (75.25 €/g) (see Supporting Information), due to the fact that the latter is prepared by means of very efficient reaction steps and using inexpensive reagents.

Finally, most of the calculated costs of compounds **1** and **8** are due to the use of not commercially available precursors, which need to be prepared and purified by chromatographic procedures (41.06 €/g and 82.64 €/g represent the total cost of purification procedures for compounds **1** and **8**, respectively). Therefore, the economic burden is brought about first by the amount and complexity of employed intermediates as reactants, secondly by chromatographic costs and only in minimal part by commercially available reactants expense (with rare exceptions). Solvents for washings also contribute in enhancing the price per gram of the target molecule.

Table 4 reports the Eco Scale calculation outcomes. According to the definition of this semi-quantitative tool, results show a less negative value of Eco Scale for the one-pot C-H activation route approach, which correlates with improved human health and ecological compatibility.

**Table 4:** Eco Scale results for the original Suzuki-Miyaura route, the C-H/Stille route and the one-pot C-H activation route.

**Synthetic route**	**Eco scale rate**
Suzuki-Miyaura	−474.59
C-H/Stille	−470.55
One-pot C-H activation	−375.13

Penalty points for any single intermediate are provided in the Supporting Information. Regarding the score of the original Suzuki-Miyaura route, it should be noted that it is highly affected by the very low overall efficiency of the whole process, despite the penalty points pertaining to compound **2**, **3**, and **4** are lower than those calculated for compounds **8** and **10**. The enhancement of 95.42 points in Eco scale between the C-H/Stille route and the *one-pot* C-H activation route represents a very interesting outcome since many common synthetic steps are shared by the two processes. This means that, according to the sensitivity of this green metric, some improvements are actually achieved in the *one-pot* C-H activation path, resulting in a decreasing of penalty points due to the lower number of reaction steps used in that process. Shortening a reaction path usually implies a reduction in the need of possible hazardous chemicals, special technical setup, temperature adjustment protocols as well as workup and purification steps. A strong contribution in this case is due to the possibility of avoiding the preparation and use of compound **10**, which results in a reduction of penalty points of a value of 56.

Low penalty points are also found for the syntheses of products **9** and **14**. Accordingly, the procedures used require few reagents, no temperature changes (the processes are carried out at room temperature) and avoid chromatographic purification, the most environmentally impacting procedure. In the case of compound **9**, a great portion of penalties is due to the use of dry chloroform and *N*-bromosuccinimide, which are both toxic chemicals, while in the case of intermediate **14** penalties come mostly from the need of using diethyl ether, a toxic and highly flammable solvent, and of cooling the reaction mixture to 0°C. All these factors negatively affect the Eco-scale score. Conversely, production of compound **8** has an acceptable environmental impact, which is once again mostly due to an efficient chromatographic purification protocol.

The most polluting processes involve the production of compounds **18**, **19**, **16**, **10** and **1**. Penalties on intermediate **18** arise from of the need of using some hazardous reagents such as toluene, 1-hexanethiol, ethyl acetate, which are toxic and flammable, and 1,10-phenanthroline and copper iodide, which are toxic and dangerous for the environment. For each of these substances, having a double hazard symbol, penalty points should be doubled, thus contributing with a value of 10. In the case of compound **19**, in addition to toluene and diethyl ether, also aniline needs to be used, which is again classified as toxic and dangerous for the environment. Need of purification by column chromatography also downgraded the sustainability of its preparation. The most environmental impacting chemical of all the procedures is a 1.6 M solution of *n*-butyllithium in hexane, which is toxic, flammable and dangerous for the environment, and is used to prepare compound **16**, thus contributing with 15 points to the overall penalties for such intermediate. The same solution was also used to prepare intermediate **10**, but in this case lower penalty point are found thanks to the elimination of purification steps. Finally, the impact found for compound **1** is mainly due to chromatographic purification steps, microwave irradiation and the need to use *n*-butanol and THF, which are both classified as toxic and flammable, as well as chloranil, which is toxic and dangerous for the environment.

The last step for the preparation of **TTZ5** has been purposely excluded in the above detailed analysis, since it is performed almost in the same way in all the three procedures. However, detailed analysis on **TTZ5** shows that most penalties do not arise from the specific conditions used in the Knoevenagel condensation, but rather from consideration on the overall yield of the synthetic sequence. Specifically, in both routes only 23 penalty points are calculated, which attests to a good Eco-scale value.

Finally, it should be considered that the lower impact of the *one-pot* C-H activation process derives not only from the lower number of reaction steps, but also from slight improvements in the synthesis of the precursor **6**, comprising optimization of work-up and purification methods, avoidance of temperature adjustments steps and use of a less complicated technical setup.

In summary, we can conclude that most of the penalties for **TTZ5** production arise from safety concerns due to the use of some hazardous reagent. This means that the substitution of some reagents with greener alternatives would mostly minimize ecotoxicological impact, according to the Eco scale metric. Further improvement of green metric should originate from a careful choice of purification processes, i.e., minimization of column chromatography procedures and reduction of the amount of washing solvents. Costs, on the other hand, do not affect sustainability to a large extent. In this context, a specific attention should be put in limiting special technical setup requirements, reaction time and temperature adjustments steps.

**Figure 3:**
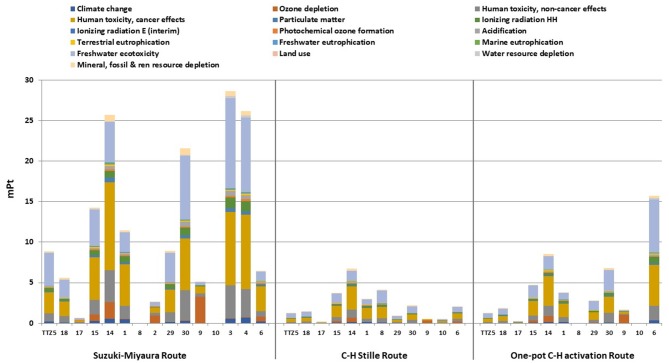
Single score results for the original and the two new procedures (LCIA method: ILCD 2011 Midpoint+ V1.10/EC-JRC Global, equal weighting). Compounds numbering on the x-axis refers to labels reported in Schemes 1, 2.

A deeper assessment of the environmental sustainability of the synthetic routes for **TTZ5** production has been accomplished through the implementation of LCA. Figure 3 reports the eco-profile single scores comparison for all the compounds generated via the two alternative synthetic routes and the original one. It is evident that the eco-profiles of reactants and compounds generated by the C-H/Stille route and the *one-pot* C-H activation route perform better than those obtained via the original Suzuki-Miyaura route. More in detail, the analysis of the diagrams immediately reveals the major issue affecting the *one-pot* C-H activation procedure: in order to obtain 1 gram of the final product **TTZ5**, the mass balance principle required in LCA calculation leads to the demand of larger quantities of reactants, chemicals and solvents. This is mainly due to the reaction yield of the penultimate step (26% for the formation of compound **6**), for which a higher global single score (15.69 mPt) is obtained compared to the corresponding result in the C-H/Stille Route (2.08 mPt) due to the need of a larger amount of aldehydic precursor. Furthermore, this trend, although less pronounced, is present along the whole compound series in the *one-pot* C-H activation path.

The analysis of any single contributions highlights that electricity, solvents and metals are the major responsible of the environmental burden on all the impact categories. For instance, concerning solvents, the largest contribution on the single score value is given by toluene, with a consumption of 37.4 kg/g for the Suzuki-Miyaura route, 9.13 kg/g for the *one-pot* C-H activation route and 5.25 kg/g for the C-H/Stille route. This outcome highlights an interesting aspect of the revision of the manufacturing path via a *one-pot* direct arylation protocol (see Scheme 1): removal of the synthetic step for the isolation of compound **8** allows saving 1.3 kg/g of toluene, but this positive effect is totally counterbalanced, and even surpassed, by the quantity of toluene required to produce compound **6** directly from **1**, via the *one-pot* C-H activation route, due to the column chromatography carried out for purification (1.5 kg/g). Similar considerations can be expressed concerning the use of catalysts (palladium complexes) that affects mainly the impact category Acidification: compared to the 4.12 · 10^−5^ kg of Pd consumed for the Suzuki-Miyaura route, a consumption of 7.94 · 10^−5^ kg is required for the *one-pot* C-H activation route, while the C-H/Stille route performs even better, as only 4.04 · 10^−5^ kg of metal are necessary.

**Figure 4:**
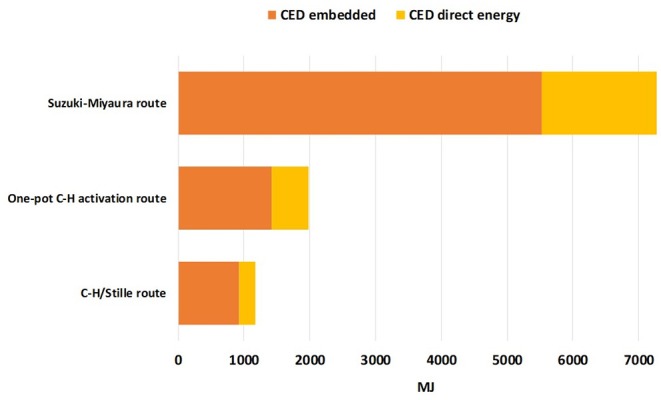
CED indicator results for the original and revised procedures.

Another important insight that can be derived from LCA calculation is the amount of direct and indirect energy consumption relative to the **TTZ5** production. In Figure 4, the CED indicator is used to show differences in terms of energy consumption between the three alternative synthetic routes. In general, a substantial improvement in the CED indicator performance is obtained compared to the original Suzuki-Miyaura route.

In particular, for the C-H/Stille route, a consumption of 246 MJ (68.33 kWh) of electricity is required per gram of final product, while the energy embedded in all raw materials used accounts for 924 MJ. For the *one-pot* C-H activation route, these values increase, with a value of 563 MJ (156.38 kWh) of electricity required per gram of final product and 1417 MJ of energy embedded in all raw materials employed (see Supporting Information). Concerning the direct energy consumption, in the *one-pot* C-H activation route a portion of almost 30% (22.7 kWh) is relative to the generation of compound **6** (which requires stirring at about 100°C for 48 h). Thus, from the LCA calculations we can claim that the difference in terms of CED between the two reaction paths is almost completely due to the global requirement of electricity and solvent (mainly toluene) which are necessary for the production of intermediate **6**. Possibly, a positive effect could be obtained if, with the same experimental setup, a larger amount of reactants and auxiliaries could be used thus producing more **TTZ5** for each reaction cycle. Indeed, a simplified sensitivity analysis on direct energy consumption conducted on the penultimate step allowed to highlight that an improvement of almost 2.5 mPt in the global single score value could be obtained if the electricity requirement for the preparation of compound **6** could be decreased up to 15 kWh.

To a further extent, the possibility to perform the LCA with primary data coming from the same synthetic procedures implemented on the pilot-scale would allow to better investigate the eco-profiles of the **TTZ5** production and to distinguish among the relevant contributions affecting the sustainability of a chemical process in an eco-design perspective.

## Conclusions

In this work, two innovative synthetic protocols for the preparation of organic dye **TTZ5**, which has been successfully employed as a sensitizer for manufacturing DSSCs, are reported. The procedures rely on two different approaches based on a C-H activation/Stille cross-coupling sequence or on a *one-pot* double C-H activation sequence and have been optimized to allow the production of **TTZ5** in gram scale. A mass-based green metrics and LCA combined approach has been employed to investigate the environmental sustainability of those two alternative synthetic routes, compared to the original **TTZ5** synthesis based on a classic Suzuki-Miyaura cross-coupling.

The comparison of the green metrics and LCA results highlights how both the new procedures allowed to complete the synthesis of the dye **TTZ5** in a more sustainable way than the previous one, considering the inferior production of waste, the lower costs and a smaller environmental impact. Despite a higher number of steps, the C-H/Stille route revealed to be more sustainable than the *one-pot* C-H activation one: this outcome is mainly highlighted by EF values and global LCA single score results (23.97 mPt and 47.35 mPt for the C-H/Stille route and the *one-pot* C-H activation route, respectively), even if the employment of toxic and/or flammable reagents such as *n*-butyllithium and tin-containing materials raised its Eco-scale value (−470.55 vs. −375.13 for the C-H/Stille route and the *one-pot* C-H activation route, respectively). In particular, the application of LCA showed that the drawback of the *one-pot* C-H activation route procedure is represented by raw material inputs for the chromatography setup: even though the number of purification operations decreases, the use of larger quantities of solvents significantly influences the environmental profile of the process, which is also strongly affected by the electricity consumption. However, despite the differences in the environmental profile of the two procedures, the possibility of carrying out the complete functionalization of TzTz **1** in just one step through two consecutive C-H activation reactions makes the *one-pot* route more attractive, from a synthetic point of view, for a lab scale preparation of **TTZ5**. In addition, such approach allows the easy and versatile synthesis of new potential thiazolothiazole-based dyes, since a rapid screening of new substituents could be easily performed by changing the donor and/or acceptor groups. However, the outcomes of the present study and, in particular, the most relevant LCA results should be carefully taken into account to guide the preparation of new photosensitizers.

The analysis performed in this work demonstrates the benefit connected with the use of LCA in environmental sustainability assessment for obtaining a trustworthy evaluation of the eco-profile of products and processes, also at lab scale. Indeed, the intrinsic less comprehensive nature of green metrics could in some cases represent a limiting factor, especially in detecting crucial issues concerning the use of resources and energy.

## Data Availability Statement

All datasets generated for this study are included in the article/Supplementary Material.

## Author Contributions

MP, AD, LZ, AM, RB, GR, and AS contributed conception and design of the study. MP, SMa, and SMo performed the green metrics and LCA analysis. AD, MC, and LZ performed the synthesis. MP, AD, and AS wrote the first draft of the manuscript. All authors contributed to manuscript revision, read and approved the submitted version.

### Conflict of Interest

The authors declare that the research was conducted in the absence of any commercial or financial relationships that could be construed as a potential conflict of interest. The reviewer SR declared a past co-authorship with one of the author SM to the handling editor.
