# Supplementary material for: Combined LCA and Green Metrics Approach for the Sustainability Assessment of an Organic Dye Synthesis on Lab Scale
Source: Front Chem. 2020 Mar 31;8:214. doi: 10.3389/fchem.2020.00214 (PMC7136579; doi:10.3389/fchem.2020.00214)
Supplement: Supplementary file 1 [file Data_Sheet_1.PDF]

frontiers

Supplementary Material

1 Experimental Section

Unless otherwise stated, all reagents were purchased from commercial suppliers and used without purification. Thiazolothiazole 1, bromide 9 and stannane 10 were prepared as previously reported [1-2]. All air-sensitive reactions were performed using Schlenk techniques. Toluene was dried on a resin exchange Solvent Purification System (MBraun) and degassed prior to use by means of the "freeze-pump-thaw" method. Petroleum ether, unless specified, is the 40-70°C boiling fraction. Reactions were monitored by TLC using silica gel 60 F254 aluminum sheet (Merck), detection was made using a KMnO4 basic solution or UV lamp. Organic phases derived from aqueous work-up were dried over Na2SO4. Flash column chromatography was performed using glass columns (10-50 mm wide) and Silica Gel 60 (230-400 mesh). 1H-NMR spectra were recorded at 400 MHz and 13C-NMR spectra were recorded at 100.6 MHz, respectively, on Bruker Avance or Varian Mercury series instruments. Chemical shifts were referenced to the residual solvent peak (CDCl3, δ 7.26 ppm for 1H-NMR and δ 77.16 ppm for 13C-NMR; C6D6, δ 7.16 ppm for 1H-NMR, δ 128.06 ppm for 13C-NMR). Coupling constants (J) were reported in Hz. ESI-MS analyses were recoded with LCQ-Fleet Ion-Trap Mass Spectrometer (Thermo). FT-IR spectra were recorded with a Perkin-Elmer Spectrum BX instrument in the range 4000-400 cm–1 with a 2 cm–1 resolution.

C-H activation/Stille-Migita route

5-(8-(5-(3,3-dipentyl-3,4-dihydro-2*H*-thieno[3,4-*b*][1,4]dioxepin-6-yl)thiazolo[5,4-*d*]thiazol-2-yl)-3,3-dipentyl-3,4-dihydro-2*H*-thieno[3,4-*b*][1,4]dioxepin-6-yl)thiophene-2-carbaldehyde (8)

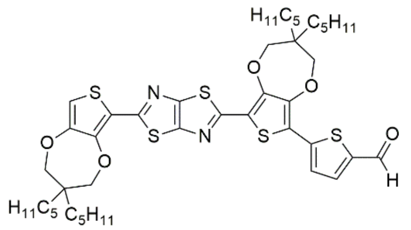

Thiazolothiazole **1** (1.04 g, 1.42 mmol, 1.0 eq.), 2-bromo-5-thiophenecarbaldehyde (**7**, 0.272 g, 1.42 mmol, 1.0 eq.), palladium acetate (0.016 g, 0.071 mmol, 5.0 mol%), CataCXium® A (0.051 g, 0.142 mmol, 10 mol%), pivalic acid (0.044 g, 0.431 mmol, 30 mol%) and potassium carbonate (0.295 g, 2.13 mmol, 1.5 eq.) were dissolved in toluene (45 mL). The resulting brown mixture was stirred at 85 °C for 20 hours, then, after cooling at room temperature, the solvent was removed. The dark orange reaction crude was purified by flash column chromatography (SiO_2; Toluene/PE 2/1, then toluene) affording pure product **8** (0.476 g, 0.566 mmol, 40% yield) as an orange solid and unreacted TzTz **1** (0.310 g, 0.424 mmol, 30% recovery).

(**8**): ^{1}H–NMR (400 MHz, $CDCl_3$): δ = 9.87 (s, 1H), 7.64 (d, *J* = 3.9 Hz, 1 H), 7.27 (d, *J* = 3.9 Hz, 1 H), 6.57 (s, 1H), 4.16 (s, 2H), 4.12 (s, 2H), 4.10 (s, 2H), 3.92 (s, 2H), 1.41–1.56 (m, 8H), 1.27–1.39 (m, 24H), 0.85–0.98 (m, 12H) ppm. ^{13}C–NMR (100 MHz, $CDCl_3$): δ = 183.0, 160.3, 158.2, 150.9, 150.6, 149.7, 148.9, 148.2, 147.3, 144.0, 142.1, 136.6, 123.8, 117.3, 117.0, 116.9, 107.4, 78.4, 78.1, 77.9, 44.2, 44.1, 32.8, 32.7, 32.2, 32.1, 22.7, 14.2 ppm.

5-(8-(5-(8-(4-(bis(4-(hexylthio)phenyl)amino)phenyl)-3,3-dipentyl-3,4-dihydro-2*H*-thieno[3,4-*b*][1,4]dioxepin-6-yl)thiazolo[5,4-*d*]thiazol-2-yl)-3,3-dipentyl-3,4-dihydro-2*H*-thieno[3,4-*b*][1,4]dioxepin-6-yl)thiophene-2-carbaldehyde (6)

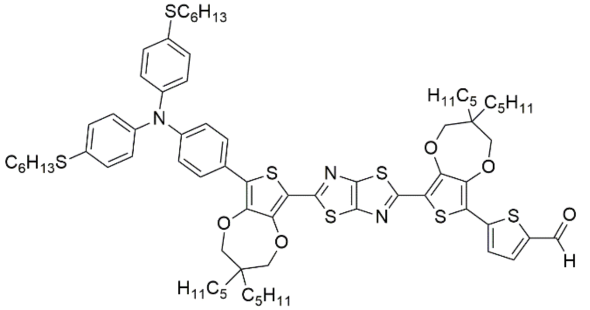

Aldehyde **8** (0.99 g, 1.18 mmol, 1.0 eq.) was dissolved in chloroform (45 mL) and acetic acid (10 mL). After cooling at –15 °C, NBS (0.210 g, 1.18 mmol, 1.0 eq.) was added in the reaction vessel. The orange solution was stirred at –15 °C for ten minutes, then the reaction was stopped by adding a saturated aqueous solution of $NaHCO_3$ (50 mL). Two phases were separated, then the organic phase was washed with brine (100 mL). After anhydrification with Na_2SO_4 and filtration, removal of the solvent afforded an orange reaction crude (1.05 g) which was used for the next step without any further purification.

^{1}H–NMR (400 MHz, $CDCl_3$): δ = 9.88 (s, 1H), 7.65 (d, *J* = 4.1 Hz, 1 H), 7.28 (d, *J* = 4.1 Hz, 1 H), 4.16 (s, 2H), 4.12 (s, 2H), 4.11 (s, 2H), 3.99 (s, 2H), 1.42–1.53 (m, 8H), 1.27–1.41 (m, 24H), 0.88–0.98 (m, 12H) ppm. ^{13}C–NMR (100 MHz, $CDCl_3$): δ = 183.0, 159.0, 158.4, 150.9, 150.6, 148.3, 147.7, 147.5, 147.3, 144.0, 142.1, 136.6, 123.8, 117.1, 116.9, 116.7, 97.2, 78.3, 78.2, 78.1, 44.2, 32.72, 32.71, 32.2, 32.0, 22.7, 22.6, 14.2 ppm.

The orange reaction crude was dissolved in toluene (60 mL), then $Pd(PPh_3)_2Cl_2$ (0.040 g, 0.057 mmol, 5 mol%) and 4-tributylstannyl-*N,N*-(4-hexylthiophenyl)aniline (**10**, 1.31 g, 1.71 mmol, 1.5 eq.) were added. The reaction mixture was heated up to 100 °C and stirred for 5 hours, then, after cooling to room temperature, the solvent was removed. Purification by flash column chromatography (SiO_2; PE/Toluene 2/1, then toluene) afforded pure product **6** (1.32 g, 1.00 mmol, 85% yield) as a black solid.

(**6**): ^{1}H–NMR (400 MHz, C_6D_6): δ = 9.57 (s, 1H), 7.82 (d, *J* = 8.7 Hz, 2H), 7.24 (d, *J* = 8.6 Hz, 4H), 7.05 (d, *J* = 8.7Hz, 2H), 6.97 (d, *J* = 8.6 Hz, 4H), 6.94 (d, *J* = 4.0 Hz, 1H), 6.87 (d, *J* = 4.0 Hz, 1H), 3.73 (s, 2H), 3.64 (s, 2H), 3.61 (s, 2H), 3.55 (s, 2H), 2.70 (t, *J* = 7.3 Hz, 4H), 1.56 (q, *J* = 7.5 Hz, 4H), 0.96–1.41 (m, 44H), 0.93 (t, *J* = 7.0 Hz, 12H), 0.85 (t, *J* = 7.0 Hz, 6H) ppm. ^{13}C–NMR (100 MHz, C_6D_6): δ = 182.2, 160.3, 158.4, 151.9, 151.4, 150.0, 148.7, 147.5, 147.4, 145.7, 143.4, 143.0, 136.1, 132.0, 131.2, 131.0, 127.4, 125.7, 125.5, 124.9, 123.8, 123.6, 117.7, 117.6, 115.0, 78.2, 78.0, 77.7, 43.8, 34.6, 33.0, 32.96, 32.4, 32.3, 31.7, 29.6, 28.8, 22.95, 22.93, 22.85, 22.78, 14.4, 14.3 ppm. IR (KBr): $\tilde{\nu}$ = 3025, 2924, 2852, 1653, 1438, 1057 cm^{-1}. ESI-MS: *m/z* = 1315.27 [M]$^+$.

C-H activation route

4-(8-(5-(3,3-dipentyl-3,4-dihydro-2*H*-thieno[3,4-*b*][1,4]dioxepin-6-yl)thiazolo[5,4-*d*]thiazol-2-yl)-3,3-dipentyl-3,4-dihydro-2*H*-thieno[3,4-*b*][1,4]dioxepin-6-yl)-*N*,*N*-bis(4-(hexylthio)phenyl)aniline (11)

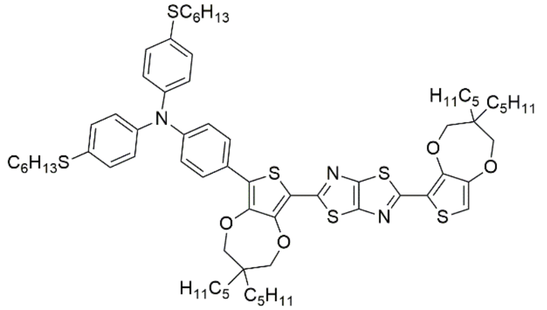

Thiazolothiazole **1** (0.189 g, 0.259 mmol, 1.5 eq.), 4-bromo-*N,N*-(4-hexylthiophenyl)aniline (**9**, 0.096 g, 0.172 mmol, 1.0 eq.), palladium acetate (1.9 mg, 8.6 μmol, 5.0 mol%), CataCXium® A (6.2 mg, 0.017 mmol, 10 mol%), pivalic acid (0.053 g, 0.052 mmol, 30 mol%) and potassium carbonate (0.036 g, 0.259 mmol, 1.5 eq.) were dissolved in toluene (5.0 mL). The resulting brown mixture was stirred at 95 °C for 6 hours, then, after cooling at room temperature, the solvent was removed. The dark orange reaction crude was purified by flash column chromatography (SiO_2; Toluene/PE 3/2, then 1/1) affording an inseparable mixture of product **11** (0.118 g, 0.098 mmol, 57% yield) and unreacted thiazolothiazole **1** (0.064 g, 0.088 mmol, 34% recovery).

(**11**): ^{1}H–NMR (400 MHz, C_6D_6): δ = 7.82 (d, *J* = 8.8 Hz, 2H), 7.24 (d, *J* = 8.2 Hz, 4H), 7.05 (d, *J* = 8.8 Hz, 2H), 6.96 (d, *J* = 8.2 Hz, 4H), 6.22 (s, 1H), 3.68 (s, 2H), 3.62 (s, 4H), 3.57 (s, 2H), 2.70 (t, *J* = 7.3 Hz, 4H), 1.55 (q, *J* = 7.4 Hz, 4H), 0.97–1.38 (m, 44H), 0.87–0.95 (m, 12H), 0.84 (t, *J* = 6.9 Hz, 6H) ppm.

(**1**): ^{1}H–NMR (400 MHz, C_6D_6): δ = 6.21 (s, 2H), 3.62 (s, 4H), 3.57 (s, 4H), 0.97–1.38 (m, 32H), 0.87–0.95 (m, 12H) ppm.

5-(8-(5-(8-(4-(bis(4-(hexylthio)phenyl)amino)phenyl)-3,3-dipentyl-3,4-dihydro-2*H*-thieno[3,4-*b*][1,4]dioxepin-6-yl)thiazolo[5,4-*d*]thiazol-2-yl)-3,3-dipentyl-3,4-dihydro-2*H*-thieno[3,4-*b*][1,4]dioxepin-6-yl)thiophene-2-carbaldehyde (6)

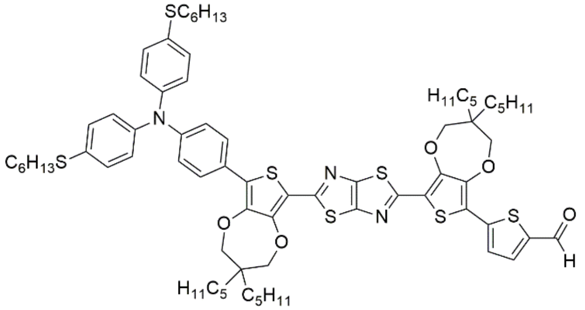

The resulting mixture of the last reaction containing intermediate **11** (0.118 g, 0.098 mmol) and thiazolothiazole **1** (0.064 g, 0.088 mmol) was dissolved in toluene (10 mL) and reacted with 2-bromo-5-thiophenecarbaldehyde (**7**, 0.052 g, 0.274 mmol, 1.0 eq.), palladium acetate (3.1 mg, 13.7 μmol, 5.0 mol%), CataCXium® A (9.8 mg, 27.4 μmol, 10 mol%), pivalic acid (8.4 mg, 0.082 mmol, 30 mol%) and potassium carbonate (0.057 g, 0.411 mmol, 1.5 eq.). The resulting brown mixture was stirred at 90 °C for 4 hours, then, after cooling at room temperature, the solvent was removed. The dark red reaction crude was purified by flash column chromatography (SiO_2; Toluene) affording pure product **6** (0.064 g, 0.049 mmol, 50% yield).

***One-pot* C-H activation route**

5-(8-(5-(8-(4-(bis(4-(hexylthio)phenyl)amino)phenyl)-3,3-dipentyl-3,4-dihydro-2*H*-thieno[3,4-*b*][1,4]dioxepin-6-yl)thiazolo[5,4-*d*]thiazol-2-yl)-3,3-dipentyl-3,4-dihydro-2*H*-thieno[3,4-*b*][1,4]dioxepin-6-yl)thiophene-2-carbaldehyde (6)

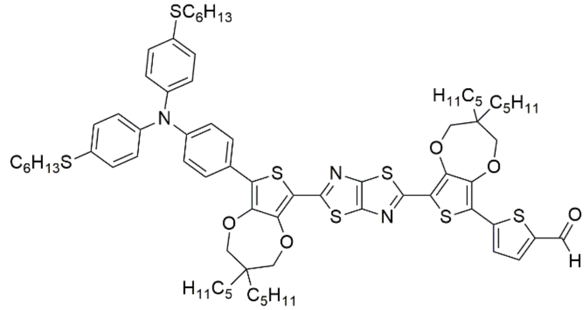

Thiazolothiazole **1** (0.50 g, 0.68 mmol, 1.0 eq.), palladium acetate (7.7 mg, 0.034 mmol, 5.0 mol%), CataCXium® A (25 mg, 0.068 mmol, 10 mol%), acetic acid (12 mg, 0.21 mmol, 12 µL, 30 mol%), potassium carbonate (0.14 g, 1.03 mmol, 1.5 eq.), 4-bromo-*N,N*-(4-hexylthiophenyl)aniline (**9**, 0.57 g, 1.03 mmol, 1.5 eq.) were dissolved in toluene (20 mL). The mixture was stirred at 95 °C for 24 hours, then again palladium acetate (7.7 mg, 0.034 mmol, 5.0 mol%), CataCXium® A (25 mg, 0.068 mmol, 10 mol%), pivalic acid (21 mg, 0.21 mmol, 30 mol%), potassium carbonate (0.14 g, 1.03 mmol, 1.5 eq.) and 2-bromo-5-thiophenecarbaldehyde (**7**, 0.26 g, 1.37 mmol, 2.0 eq.) were added to the reaction mixture, which was heated up to 110 °C. After additional 24 hours, the reaction mixture was cooled down to room temperature, diluted with toluene (100 mL) and filtered over Celite®. After evaporation of the solvent, the reaction crude was purified by flash column chromatography (SiO_2, Toluene/PE = 1/1 → 2/1 → 4/1, then toluene), which afforded the pure product **6** (0.24 g, 0.18 mmol, 26% yield) as a dark red solid.

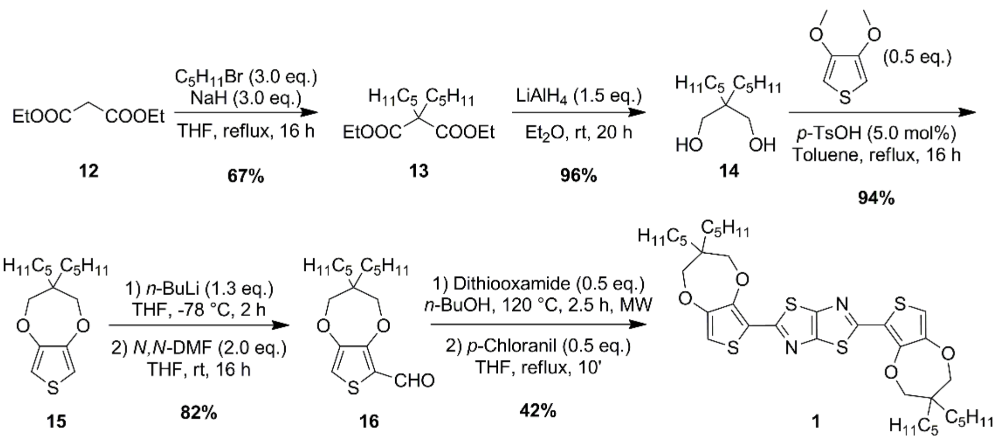

Supplementary Scheme S1. Preparation of the thiazolothiazole-based spacer **1**.

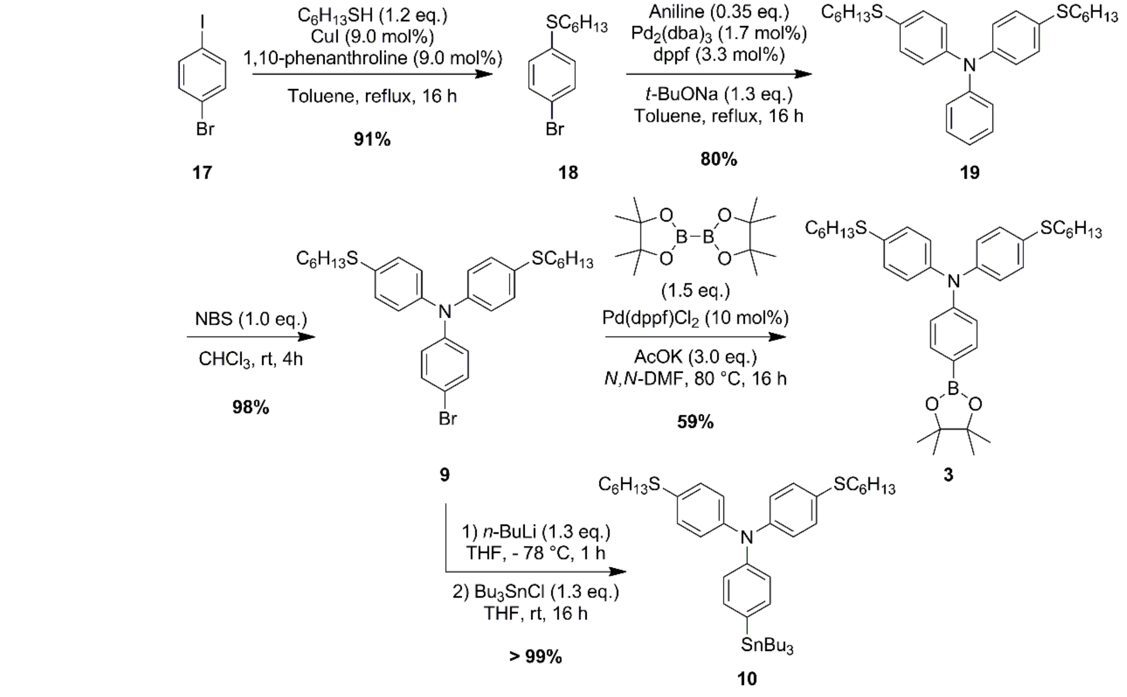

Supplementary Scheme S2. Preparation of the donor groups **3** and **10**.

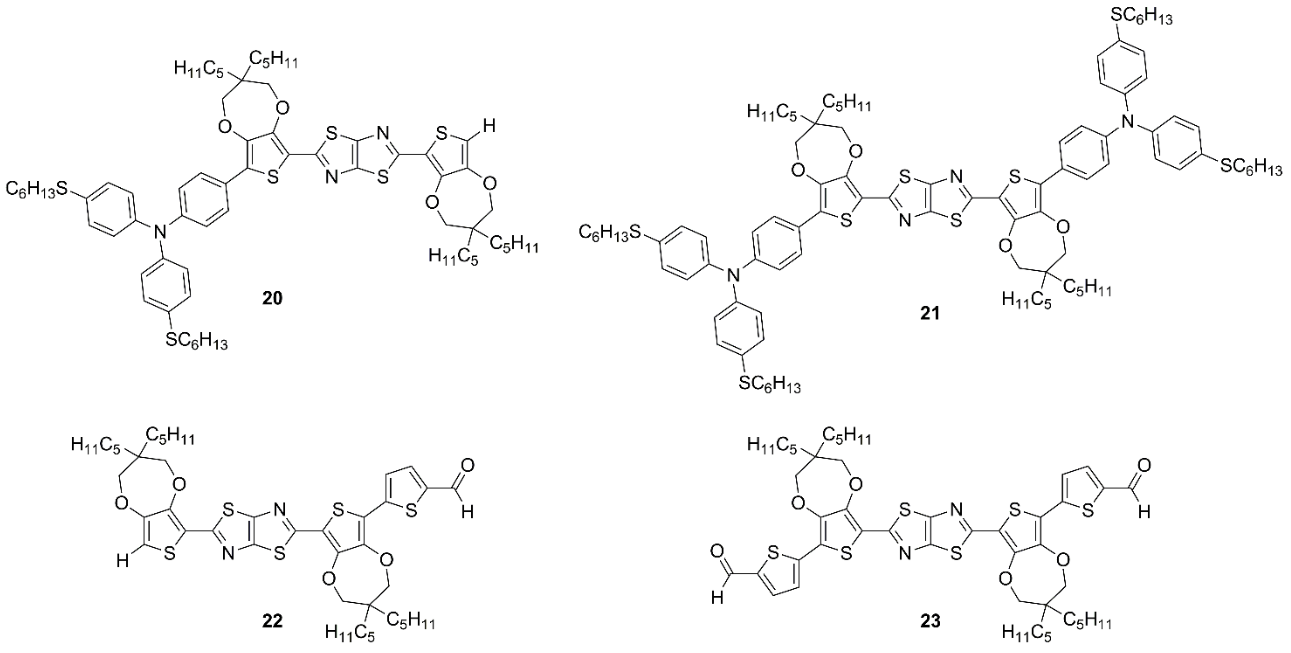

Supplementary Scheme S3. Structures of undesired coproducts.

[1] Dessì, A.; Calamante, M.; Mordini, A.; Peruzzini, M.; Sinicropi, A.; Basosi, R.; Fabrizi de Biani, F.; Taddei, M.; Colonna, D.; Di Carlo, A.; Reginato G.; Zani, L. *Chem. Commun.* **2014**, *50*, 13952.

[2] Dessì, A.; Bartolini, M.; Calamante, M.; Zani, L.; Mordini, A.; Reginato, G. *Synthesis* **2018**, *50*, 1284.

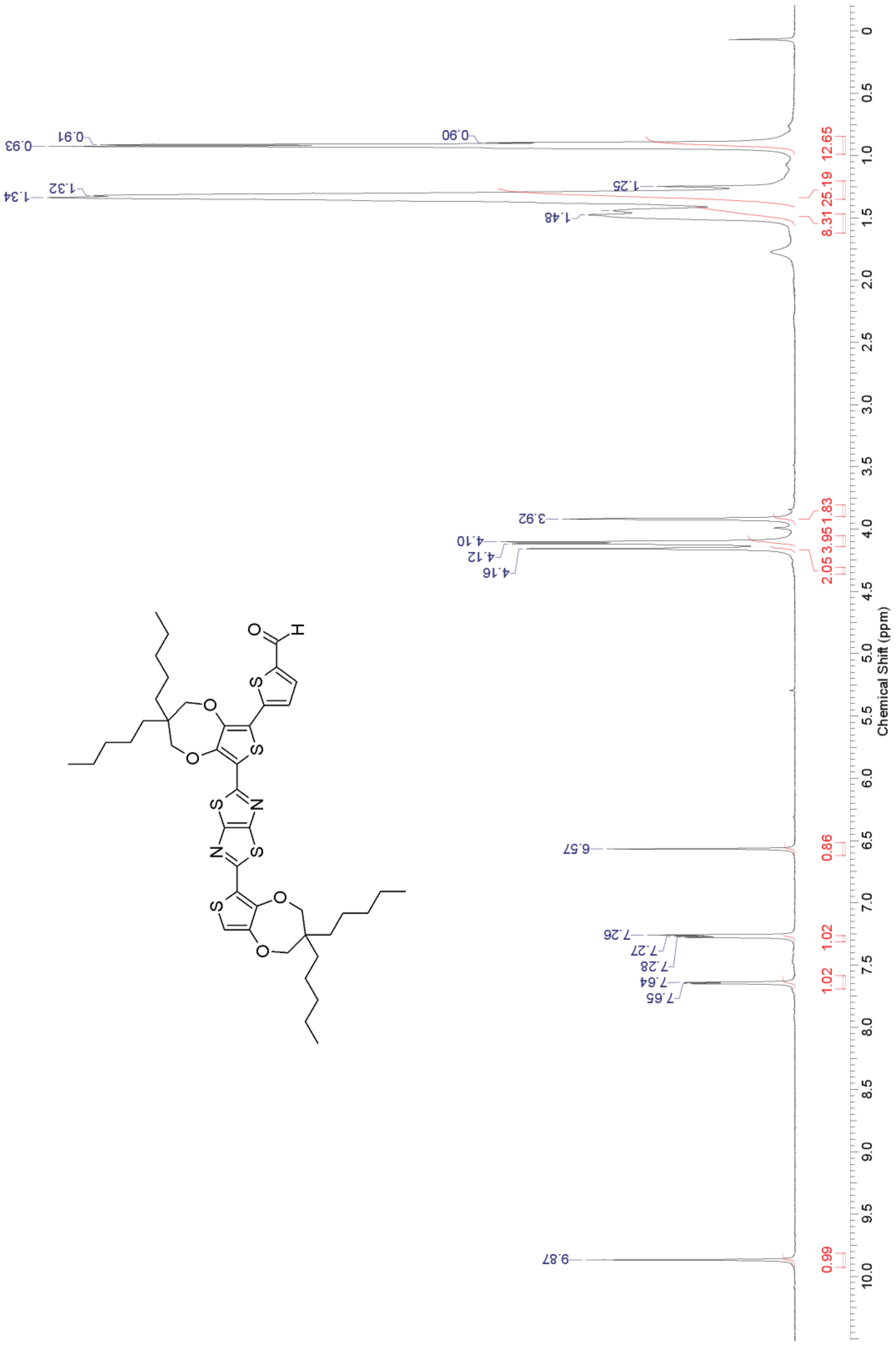
Chemical Shift (ppm)
0.90
0.91
0.93
1.25
1.32
1.34
1.48
3.92
4.10
4.12
4.16
6.57
7.26
7.27
7.28
7.64
7.65
9.87
12.65
25.19
8.31
1.83
3.95
2.05
0.86
1.02
1.02
0.99
S
N
O
H

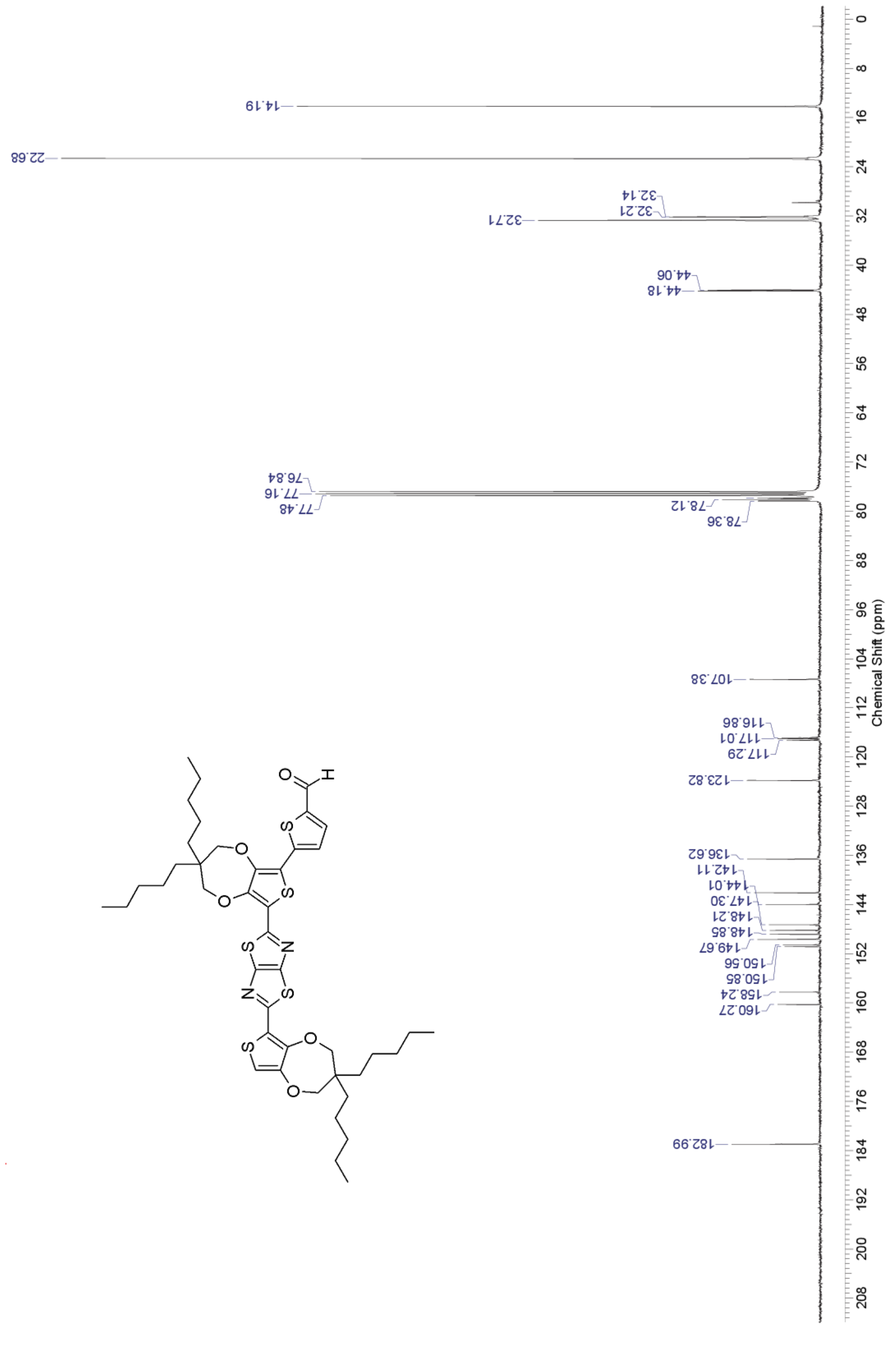
14.19
22.68
32.14
32.21
32.71
44.06
44.18
76.84
77.16
77.48
78.12
78.36
107.38
116.86
117.01
117.29
123.82
136.62
142.11
144.01
147.30
148.21
148.85
149.67
150.56
150.85
158.24
160.27
182.99
Chemical Shift (ppm)

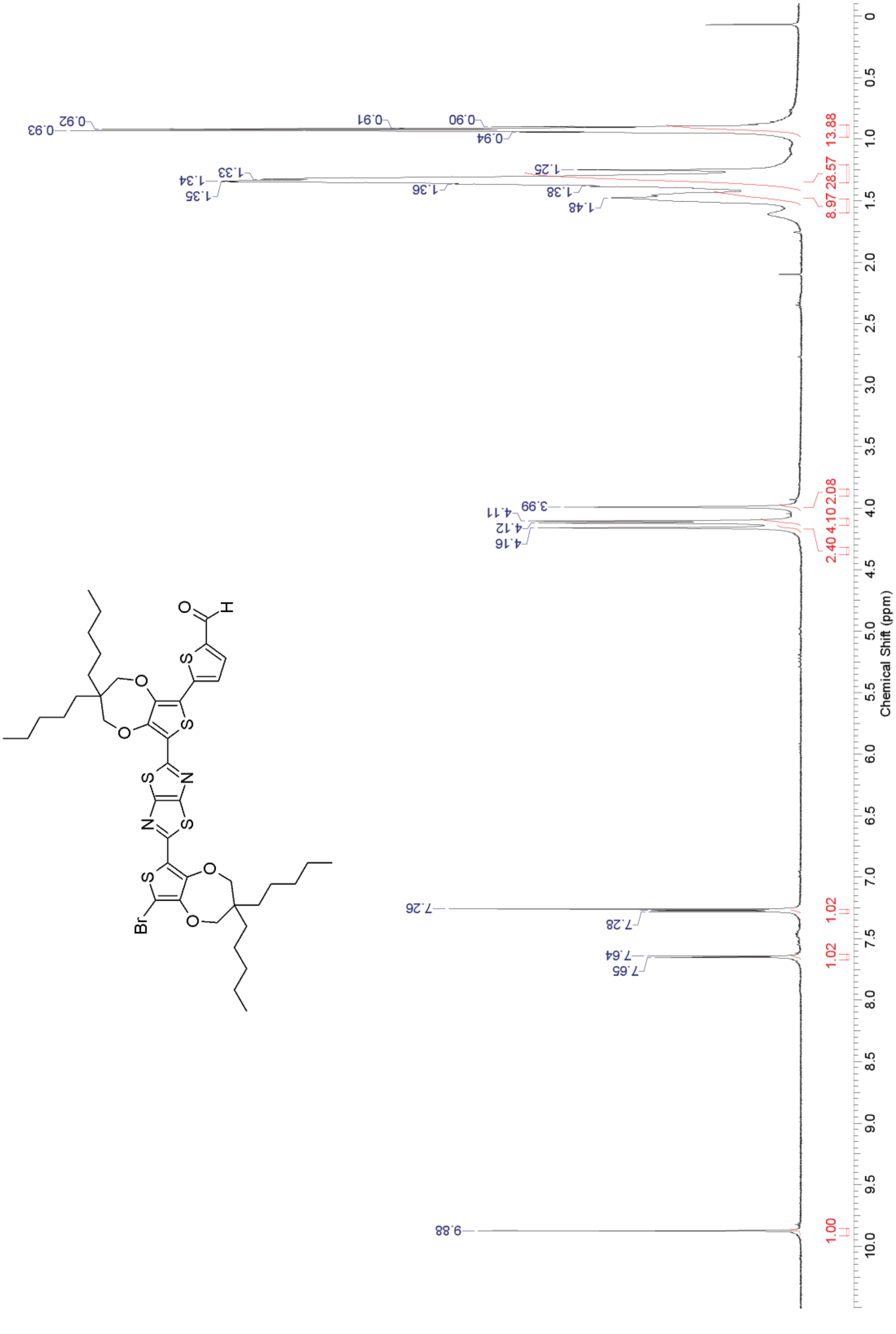
Chemical Shift (ppm)
13.88
28.57
8.97
2.08
4.10
2.40
1.02
1.02
1.00
0.93
0.92
0.91
0.90
0.94
1.25
1.33
1.34
1.35
1.36
1.38
1.48
3.99
4.11
4.12
4.16
7.26
7.28
7.64
7.65
9.88
O
H
S
N
Br

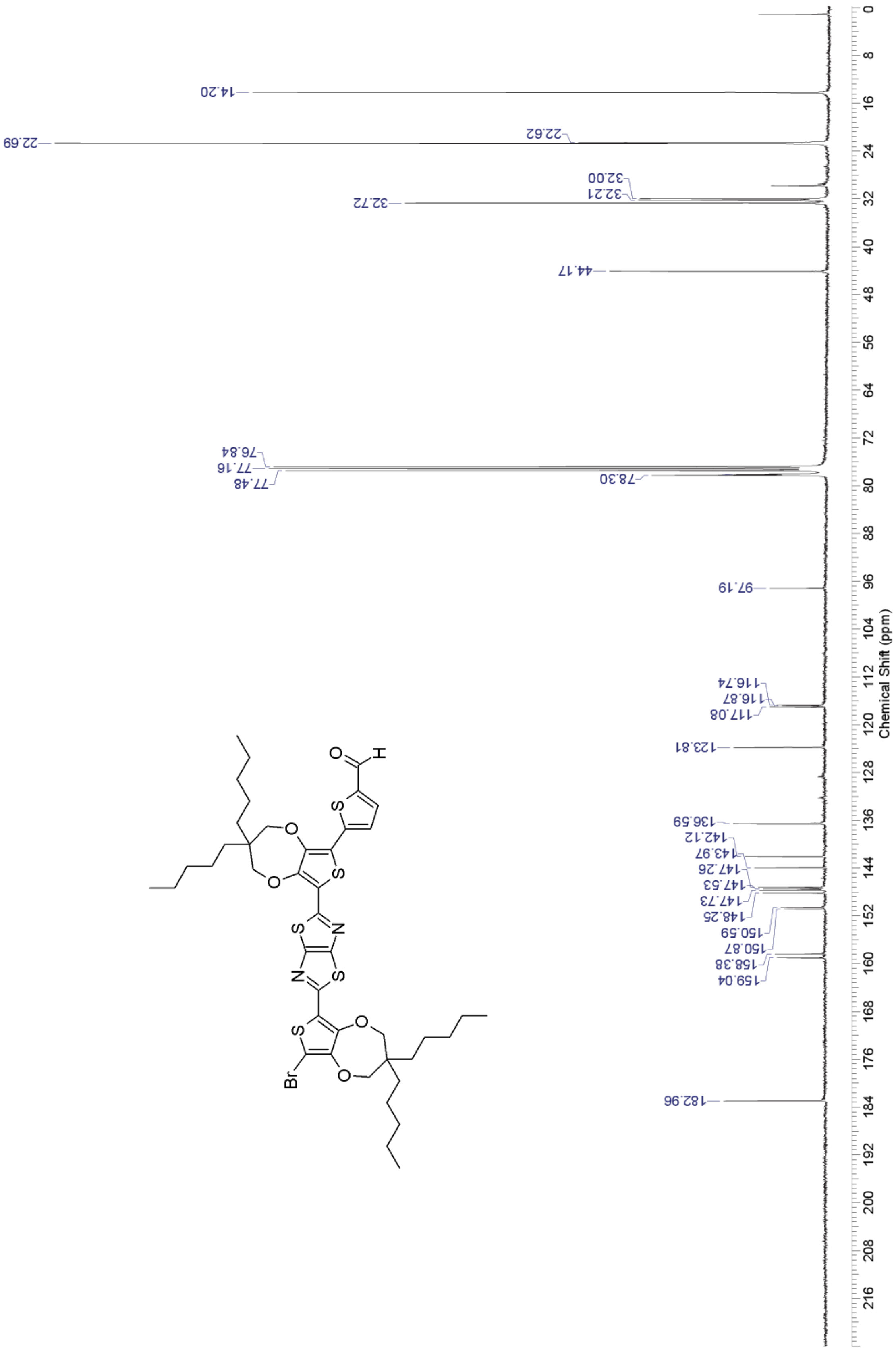

14.20
22.69
22.62
32.00
32.21
32.72
44.17
76.84
77.16
77.48
78.30
97.19
116.74
116.87
117.08
123.81
136.59
142.12
143.97
147.26
147.53
147.73
148.25
150.59
150.87
158.38
159.04
182.96
Chemical Shift (ppm)

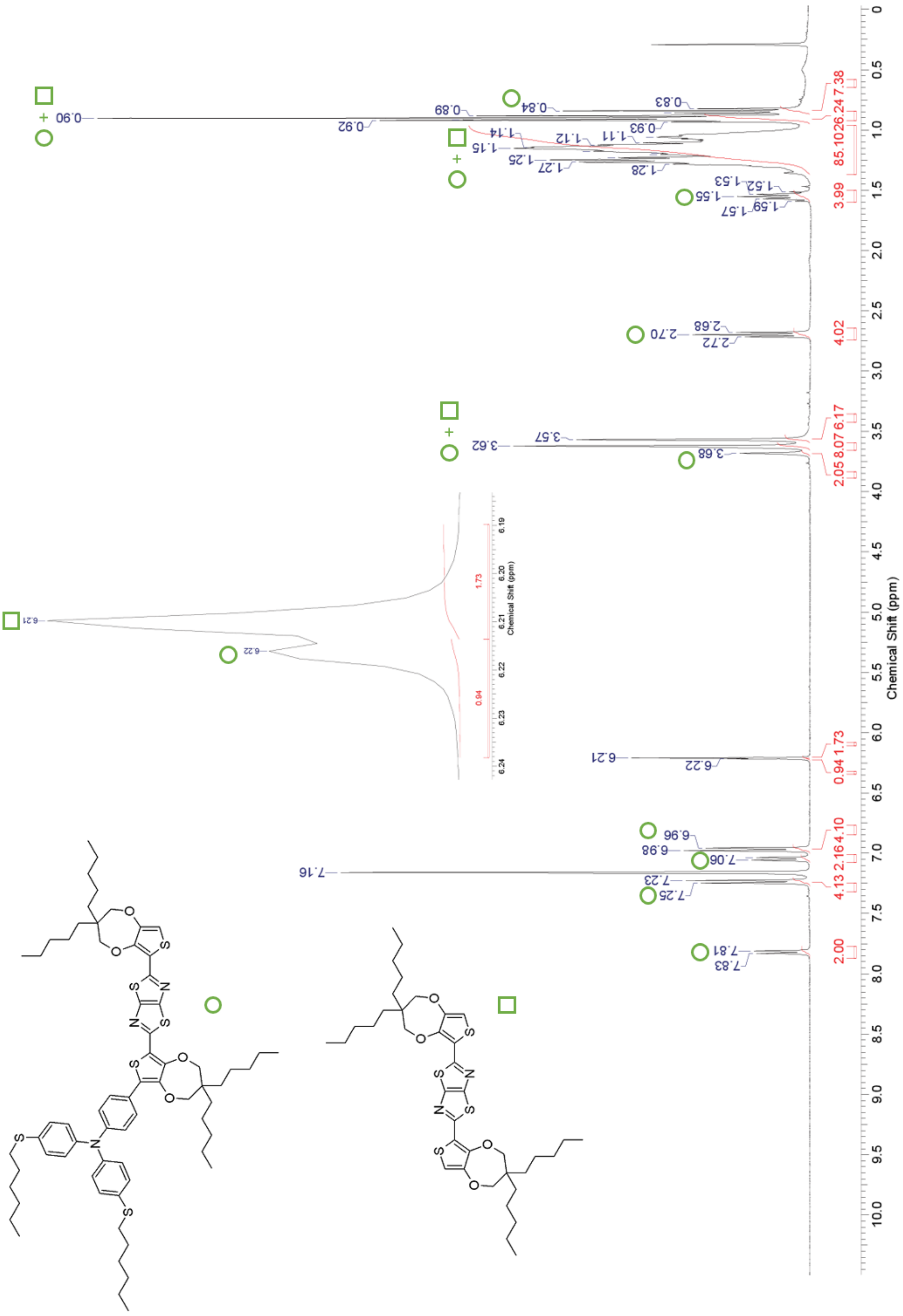
Chemical Shift (ppm)

2 Green Metrics

2.1 E-factor and costs estimates

Diethyl 2,2-dipentylmalonate (13)

Reagent	Amount (g)	Amount (mL)	Density (g/mL)	Total mass (g)	Price (€/g or €/mL)	Total price (€)
1-bromopentane	34.9			34.9	0.27	9.49
THF		200	0.89	178	0.06	11.76
Sodium hydride	5.54			5.54	0.361	1.99
Diethyl malonate	12.3			12.3	0.07	0.86
Et_2O (150 mLx2)		300	0.71	213.9	0.05	14.76
Na_2SO_4	2			2	0.03	0.06
Water (Neutralization)		30	1	30		0
1-bromopentane RECOVERED By distillation (-)	5			5	0.272	1.36

Amount **13**	15.6 g
Total waste (Water excluded)	426.04 g
Total waste (Water included)	456.04 g
E factor (Water excluded)	27.31
E factor (Water included)	29.23
Total cost	37.57 €
Cost for **13**	2.41 €/g

2,2-dipentylpropan-1,3-diol (14)

Reagent	Amount (g)	Amount (mL)	Density (g/mL)	Total mass (g)	Price (€/g or €/mL)	Total price (€)
Lithium aluminum hydride	2.95			2.95	1.6	4.72
Et_2O		100	0.713	71.3	0.05	4.92
Compound **13**	15.6			15.6	2.41	37.57
NaOH (15% of 3 mL)	0.45			0.45	0.03	0.013
Na_2SO_4 (reaction+drying)	12			12	0.03	0.36
Water (Addition+NaOH aqueous solution)		15	1	15	0	0

Amount **14**	10.8 g
Previous waste **13** (Water excluded)	426.04 g
Previous waste **13** (Water included)	456.04 g
Total waste (Water excluded)	517.54 g
Total waste (Water included)	562.54 g
E factor (Water excluded)	47.92
E factor (Water included)	52.09
Total cost	47.59 €
Cost for **14**	4.41 €/g

3,3-dipentyl-3,4-dihydro-2*H*-thieno[3,4-*b*][1,4]dioxepine (ProDOT, 15)

Reagent	Amount (g)	Amount (mL)	Density (g/mL)	Total mass (g)	Price (€/g or €/mL)	Total price (€)
3,4-dimethoxythiophene	1.67			1.67	11.74	19.61
Toluene		100	0.87	86.7	0.04	3.58

Compound **14**	5			5	4.41	22.03
p-toluenesulfonic acid	0.22			0.22	0.14	0.03
Na_2SO_4	2			2	0.030	0.06
Silica cartridge (column chromatography)		125,6	2.6	326.56	0.03	10.45
Petroleum ether 40-60 °C		600	0.65	390	0.03	19.68
Dichloromethane		400	1.33	532	0.03	10.16
Ethyl acetate		200	0.90	180.4	0.03	6.34
Water (Washing)		100	1	100		0
Recovered **14** through column chromatography	1.99			1.99	4.41	8.77

Amount **15**	3.22 g
Previous waste **14** (Water excluded)	239.60 g
Previous waste **14** (Water included)	260.44 g
Total waste (Water excluded)	1811.24 g
Total waste (Water included)	1932.08 g
E factor (Water excluded)	562.50
E factor (Water included)	600.02
Total cost	83.17 €
Cost for **15**	25.83 €/g

3,3-dipentyl-3,4-dihydro-2*H*-thieno[3,4-*b*][1,4]dioxepine-6-carboxaldehyde (16)

**Reagent**	**Amount (g)**	**Amount (mL)**	**Density (g/mL)**	**Total mass (g)**	**Price (€/g or €/mL)**	**Total price (€)**
ProDOT **15**	1.09			1.09	25.83	28.15
THF		30	0.89	26.7	0.059	1.76
1,6 M solution of *n*-BuLi in hexane		3	0.68	2.04	0.58	1.75

N,*N*-DMF	0.538			0.538	0.37	0.20
NH_4Cl		50	1.53	76.5	0.048	2.42
Et_2O (extraction)		100	0.713	71.3	0.049	4.92
Na_2SO_4(drying)	1			1	0.030	0.03
Silica cartridge (column chromatography)		100.48	2.6	261.248	0.032	8.34
Petroleum ether 40-60 °C		600	0.65	390	0.033	19.68
Dichloromethane		300	1.37	411	0.025	7.62
Water (aqueous solution of NH4Cl)		50	1	50		0
ProDOT **15** recuperato mediante colonna cromatografica (-)	0.1			0.1	25.83	2.59

Amount **16**	0.972 g
Previous waste **15** (Water excluded)	613.12 g
Previous waste **15** (Water included)	654.03 g
Total waste (Water excluded)	1853.46 g
Total waste (Water included)	1944.37 g
E factor (Water excluded)	1906.86
E factor (Water included)	2000.38
Total cost	72.32 €
Cost for **16**	74.4 €/g

ProDOT-TzTz (1)

**Reagent**	**Amount (g)**	**Amount (mL)**	**Density (g/mL)**	**Total mass (g)**	**Price (€/g or €/mL)**	**Total price (€)**
Dithiooxamide	0.106			0.106	11.44	1.21

Compound **16**	0.57			0.57	74.39	42.41
n-butanol		1.5	0.81	1.215	0.06	0.08
THF		5	0.89	4.45	0.0588	0.29
chloranil	0.108			0.11	0.25	0.027
Silica cartridge (column chromatography)		84.84	2.6	220.58	0.032	7.06
Petroleum ether 40-60 °C		600	0.65	390	0.0328	19.68
Toluene		400	0.867	346.8	0.0358	14.32

Amount **1**	0.269 g
Previous waste **16** (Water excluded)	1086.91 g
Previous waste **16** (Water included)	1140.22 g
Total waste (Water excluded)	2091.03 g
Total waste (Water included)	2144.33 g
E factor (Water excluded)	7773.36
E factor (Water included)	7971.50
Total cost	85.27 €
Cost for **1**	316.29 €/g

2,5-bis(8-iodo-3,3-dipentyl-3,4-dihydro-2*H*-thieno[3,4-*b*][1,4]dioxepin-6-yl)thiazolo [5,4-*d*]thiazole (2)

**Reagent**	**Amount (g)**	**Amount (mL)**	**Density (g/mL)**	**Total mass (g)**	**Price (€/g or €/mL)**	**Total price (€)**
Compound 1	2.03			2.03	316.29	643.50
N-iodosuccinimide	1.56			1.56	4.63	7.223
dry $CHCl_3$	0.161	50	1.492	74.6	0.057	2.85

Acetic Acid	0.648	50	1.05	52.5	0.422	21.1
methanol		100	0.792	79.2	0.0204	2.044
ethyl acetate	3	100	0.902	90.2	0.0317	3.17
Amount **2**			2.53 g			
Previous waste **1** (Water excluded)			15779.87 g			
Previous waste **1** (Water included)			16182.15 g			
Total waste (Water excluded)			16077.43 g			
Total waste (Water included)			16479.71 g			
E factor (Water excluded)			6354.72			
E factor (Water included)			6513.72			
Total cost			679.88 €			
Cost for **2**			268.73 €/g			

Extended ProDOT-TzTz-aldehyde (8)

**Reagent**	**Amount (g)**	**Amount (mL)**	**Density (g/mL)**	**Total mass (g)**	**Price (€/g or €/mL)**	**Total price (€)**
Compound **1**	1.04			1.04	316.29	329.67
2-bromo-5-thiophenecarbaldehyde	0.272			0.272	1.58	0.43
palladium acetate	0.016			0.016	21	0.34
CataCXium A	0.051			0.051	75.4	3.85
pivalic acid	0.044			0.044	0.257	0.01
potassium carbonate	0.295			0.295	0.1424	0.042
toluene		45	0.867	39.015	0.0358	1.611
Silica cartridge (column chromatography)		150.72	2.6	391.87	0.032	12.54

Petroleum ether 40-60 °C		500	0.65	325	0.03	16.4
Toluene (chromatography)		1500	0.867	1300.50	0.04	53.7
Unreacted TzTz **1** COPRODUCT	0.31			0.31	0	0
COPRODUCT **23**	0.2			0.2	0	0

Amount **8**	0.476 g
Previous waste **1** (Water excluded)	8084.27 g
Previous waste **1** (Water included)	8290.36 g
Total waste (Water excluded)	10142.41 g
Total waste (Water included)	10348.50 g
E factor (Water excluded)	21307.58
E factor (Water included)	21740.55
Total cost	418.59 €
Cost for **8**	879.39 €/g

1-bromo-4-hexylthiobenzene (18)

**Reagent**	**Amount (g)**	**Amount (mL)**	**Density (g/mL)**	**Total mass (g)**	**Price (€/g or €/mL)**	**Total price (€)**
1-bromo-4-iodobenzene (**17**)	5.66			5.66	0.54	3.06
Toluene		55	0,867	47.69	0.034	1.97
Copper iodide	0.34			0.34	0.19	0.06
1,10-phenanthroline	0.324			0.324	1.35	0.44
sodium *tert*-butoxide	2.88			2.88	0.40	1.16
1-hexanethiol	2.84			2.84	0.88	2.51
ethyl acetate		150	0,902	135.3	0.04	4.76

Silica cartridge (column chromatography)		197,82	2,6	514.33	0.03	16.44
Petroleum ether 40-60 °C		2000	0,65	1300	0.033	65.6
1,2-dihexyldisulfane COPRODUCT	0.2			0.2	0	0
Non reacted substrate COPRODUCT	0.25			0.25	0	0
**17** RECOVERED through column chromatography	0.25			0.25	0.54	0.135
Amount **18**	4.94 g					
Total waste (Water excluded)	2004.62					
Total waste (Water included)	2004.62					
E factor (Water excluded)	405.79					
E factor (Water included)	405.79					
Total cost	95.87 €					
Cost for **18**	19.41 €/g					

4-(Hexylthio)-*N*-(4-(hexylthio)phenyl)-*N*-phenylaniline (19)

**Reagent**	**Amount (g)**	**Amount (mL)**	**Density (g/mL)**	**Total mass (g)**	**Price (€/g or €/mL)**	**Total price (€)**
**18** (1-Bromo-4-hexylthiobenzene)	4.74			4.74	19.41	91.99
Toluene		55	0.867	47.66	0.036	1.97
$Pd_2(dba)_3.CHCl_3$	0.3			0.3	9	2.7
1,1'-bis-(diphenylphosphino)ferrocene	0.321			0.32	1.13	0.36
aniline	0.539			0.54	0.274	0.15

sodium *tert*-butoxide	2.22			2.22	0.40	0.89
Et_2O		200	0.731	146.2	0.049	9.84
Na_2SO_4	2			2	0.030	0.06
Silica cartridge (column chromatography)		226.08	0.65	146.95	0.03	4.70
Petroleum ether 40-60 °C		2500	0.65	1625	0.03	82
Toluene (highly flammable) (chromatography)		500	0.867	433.5	0.04	17.9
4-(hexylthio)-N-phenylaniline COPRODUCT	0.16			0.16	0	0
1-bromo-4-hexylthiobenzene non reacted COPRODUCT	1			1	19.41	19.41
Water (washing)		150	1	150	0	0
**18** RECOVERED through column cromatography (-)	1			1	19.41	19.41

Amount **19**	2.22 g
Previous waste **18** (Water excluded)	1923.46 g
Previous waste **18** (Water included)	1923.46 g
Total waste (Water excluded)	4330.86 g
Total waste (Water included)	4480.86 g
E factor (Water excluded)	1950.84
E factor (Water included)	2018.41
Total cost	212.57 €
Cost for **19**	95.75 €/g

4-Bromo-*N*,*N*-bis(4-(hexylthio)phenyl)aniline (9)

Reagent	Amount (g)	Amount (mL)	Density (g/mL)	Total mass (g)	Price (€/g or €/mL)	Total price (€)
Compound **19**	1..53			1,53	95,75	146,50
dry $CHCl_3$		30	1,492	44,76	0,06	1,71
N-bromosuccinimide	0..569			0,569	0,16	0,09
$CHCl_3$		100	1,492	149,2	0,06	5,7
Na_2SO_4	2			2	0,03	0,06
Water (washing)		150	1	150	0	0
Amount **9**			1.75 g			
Previous waste **19** (Water excluded)			2984.78 g			
Previous waste **19** (Water included)			3088.16 g			
Total waste (Water excluded)			3181.09 g			
Total waste (Water included)			3434.47 g			
E factor (Water excluded)			1817.77			
E factor (Water included)			1962.55			
Total cost			154.06 €			
Cost for **9**			88.03 €/g			

4-(Hexylthio)-*N*-(4-(hexylthio)phenyl)-*N*-(4-(4,4,5,5-tetramethyl-1,3,2-dioxaborolan-2-yl)phenyl)aniline (3)

Reagent	Amount (g)	Amount (mL)	Density (g/mL)	Total mass (g)	Price (€/g or €/mL)	Total price (€)
Compound **9**	1.23			1,23	88,035	108,283
Bis(pinacolato)diboron	0.838	30		0,838	0,442	0,37

Pd(dppf)Cl_2	0.161			0,161	10,51	1,69
Potassium acetate	0.648	100		0,648	0,065	0,042
N,*N*-DMF		22	0,944	20,768	0,368	8,09
Na_2SO_4 (drying)	3			3	0,030	0,091
diethyl ether		200	0,713	142,6	0,049	9,84
Water (washing)		450	1	450		
Silica cartridge (column chromatography)		226	0,65	146,9	0,032	7,23
Petroleum ether 40-60 °C		1000	0,65	650	0,033	32,8
Toluene (highly flammable) (chromatography)		1500	0,867	1300,5	0,036	53,7
Amount **3**			0.788 g			
Previous waste **9** (Water excluded)			2235.85 g			
Previous waste **9** (Water included)			2413.94 g			
Total waste (Water excluded)			4501.71 g			
Total waste (Water included)			5129.80 g			
E factor (Water excluded)			5712.83			
E factor (Water included)			6509.90			
Total cost			222.15 €			
Cost for **3**			281.91 €/g			

4-(Hexylthio)-*N*-(4-(hexylthio)phenyl)-*N*-(4-(8-(5-(8-iodo-3,3-dipentyl-3,4-dihydro-2*H*-thieno[3,4-*b*][1,4]dioxepin-6-yl)thiazolo[5,4-*d*]thiazol-2-yl)-3,3-dipentyl-3,4-dihydro-2*H*-thieno[3,4-*b*][1,4]dioxepin-6-yl)phenyl)aniline (4)

Reagent	Amount (g)	Amount (mL)	Density (g/mL)	Total mass (g)	Price (€/g or €/mL)	Total price (€)

Compound **2**	0.75			0,99	268,73	266,04
Compound **3**	0.461			0,056	281,91	15,787
Pd(dppf)Cl_2	0.056	50	1,492	0,056	10,51	0,589
KF	0.222	50	1,05	0,222	0,163	0,0362
Toluene		15	0,867	13,005	0,0358	0,537
Ethanol	3	15	0,789	11,835	0,0188	0,282
Silica cartridge (column chromatography)		236	0,65	153,4	0,032	7,552
Petroleum ether 40-60 °C		1000	0,65	650	0,0328	32,8
Toluene		1500	0,867	1300,5	0,0358	53,7
Compound **2** recovered	0.495			0,495	117,853	58,34
Amount **4**			0.178 g			
Previous waste **2** (Water excluded)			6291.17 g			
Previous waste **2** (Water included)			6448.58 g			
Previous waste **3** (Water excluded)			319.92 g			
Previous waste **3** (Water included)			364.55 g			
Total waste (Water excluded)			8740.48 g			
Total waste (Water included)			8942.53 g			
E factor (Water excluded)			49103.81			
E factor (Water included)			50238.93			
Total cost			318.99 €			
Cost for **4**			1792.07 €/g			

4-Tributylstannyl-*N*,*N*-(4-hexylthiophenyl)aniline (10)

Reagent	Amount (g)	Amount (mL)	Density (g/mL)	Total mass (g)	Price (€/g or €/mL)	Total price (€)
Compound **9**	0.2			0.2	88.03	17.61
THF		2	0.89	1.78	0.059	0.12
1.6 M solution of *n*-BuLi in hexane		0.29	0.68	0.19	0.58	0.17
Bu_3SnCl	0.152			0.15	0.37	0.056
ethyl acetate		50	0.902	45.1	0.03	1.585
Na_2SO_4 (drying)	1			1	0.03	0.03
Water (washing)		150	1	150	0	0

Amount **10**	0.26 g
Previous waste **9** (Water excluded)	363.55 g
Previous waste **9** (Water included)	392.51 g
Total waste (Water excluded)	411.72 g
Total waste (Water included)	590.68 g
E factor (Water excluded)	1583.55
E factor (Water included)	2271.85
Total cost	19.57 €
Cost for **10**	75.25 €/g

TTZ5 precursor aldehyde (6) "SUZUKI-MIYAURA ROUTE" SYNTHESIS

Reagent	Amount (g)	Amount (mL)	Density (g/mL)	Total mass (g)	Price (€/g or €/mL)	Total price (€)
Compound **4**	0.16			0.16	1792.07	286.73
5-Formylthiophene-2-boronic acid	0.035			0.035	3.35	0.117

$Pd(dppf)Cl_2$	0.0044			0.0044	10.51	0.0462
KF	0.042			0.042	0.163	0.0068
Na_2SO_4 (drying)	2			2	0.0302	0.0604
Toluene	0.57	5.5	0.867	4.7685	0.0358	0.1969
Methanol		2.75	0.792	2.178	0.02044	0.0562
Silica cartridge (column chromatography)	0.0077	9.4	0.65	6.11	0.032	0.301
Toluene (chromatography)	0.025	600	0.867	520.2	0.0358	21.48
Dichloromethane	0.021	100	1.327	132.7	0.057	5.7

Amount aldehydic precursor **6**	0.09 g
Previous waste 4 (Water excluded)	7854.61 g
Previous waste 4 (Water included)	8038.23 g
Total waste (Water excluded)	8524.72 g
Total waste (Water included)	8706.34 g
E factor (Water excluded)	94719.08
E factor (Water included)	96737.07
Total cost	314.70 €
Cost for aldehydic precursor **6**	3496.62 €/g

TTZ5 precursor aldehyde (6) PART A “C-H/STILLE ROUTE” SYNTHESIS

**Reagent**	**Amount (g)**	**Amount (mL)**	**Density (g/mL)**	**Total mass (g)**	**Price (€/g or €/mL)**	**Total price (€)**
Compound **8**	0.99			0.99	879.39	870.60
chloroform		45	1.492	67.14	0.06	2.565

acetic acid		10	1.049	10.49	0.42	4.22
NBS	0.21			0.21	0.16	0.03
$NaHCO_3$		50	2.16	108	0.03	1.44
Na_2SO_4	2			2	0.03	0.06
Water ($NaHCO_3$ saturated solution)		50	1	50	0	0
Amount **orange reaction crude**			1.05 g			
Previous waste 8 (Water excluded)			20684.93 g			
Previous waste 8 (Water included)			21113.57 g			
Total waste (Water excluded)			20872.71 g			
Total waste (Water included)			21351.35 g			
E factor (Water excluded)			19878.77			
E factor (Water included)			20334.62			
Total cost			878.91 €			
Cost for **orange reaction crude**			837.06 €/g			

TTZ5 precursor aldehyde (6) PART B “C-H/STILLE ROUTE” SYNTHESIS

**Reagent**	**Amount (g)**	**Amount (mL)**	**Density (g/mL)**	**Total mass (g)**	**Price (€/g or €/mL)**	**Total price (€)**
orange reaction crude	1.05			1.05	837.06	889.37
Toluene		60	0.867	52.02	0.036	2.15
$Pd(PPh_3)_2Cl_2$	0.04			0.04	9.03	0.3612
Compound **10**	1.31			1.31	75.25	100.33
Silica cartridge (column chromatography)		113.04	2.3	259.99	0.032	8.32
Petroleum ether 40-60 °C		1000	0.65	650	0.03	32.8
Toluene (highly flammable) (chromatography)		1000	0.867	867	0.04	35.8

Water (washing)		150	1	150	0	0
Amount aldehydic precursor **6**	1.32 g					
Previous waste orange reaction crude (Water excluded)	21282.28 g					
Previous waste orange reaction crude (Water included)	21760.93 g					
Previous waste **10** (Water excluded)	2074.45 g					
Previous waste **10** (Water included)	2976.12 g					
Total waste (Water excluded)	25186.83 g					
Total waste (Water included)	26717.14 g					
E factor (Water excluded)	19080.93					
E factor (Water included)	20240.26					
Total cost	1056.92€					
Cost for aldehydic precursor **6**	800.70 €/gr					

TTZ5 precursor aldehyde (6) "ONE-POT C-H ACTIVATION ROUTE" SYNTHESIS

**Reagent**	**Amount (g)**	**Amount (mL)**	**Density (g/mL)**	**Total mass (g)**	**Price (€/g or €/mL)**	**Total price (€)**
Compound **1**	0.5			0.5	316.29	158.15
palladium acetate	0.0077			0.0077	21	0.16
CataCXium® A	0.025			0.025	75.4	1.89
acetic acid	0.012			0.012	0.42	0.01
potassium carbonate	0.14			0.14	0.142	0.02
Compound **9**	0.57			0.57	85.20	48.57
anhydrous toluene		20	0.867	17.34	0.04	0.72
palladium acetate	0.0077			0.0077	21	0.16
CataCXium® A	0.025			0.025	75.4	1.89

pivalic acid	0.021			0.021	0.26	0.01
potassium carbonate	0.14			0.14	0.14	0.02
5-bromo-2-thiophenecarboxaldehyde	0.26			0.26	1.58	0.41
Toluene		100	0.867	86.7	0.04	3.58
Silica cartridge (column chromatography)		106.05	2.6	275.73	0.03	8.82
Petroleum ether 40-60 °C		500	0.65	325	0.04	16.4
Toluene (chromatography)		1500	0.867	1300.5	0.04	53.7
COPRODUCT **20**	0.055			0.055	0	0
COPRODUCT **21**	0.075			0.075	0	0
COPRODUCT **22**	0.121			0.121	0	0
COPRODUCT **23**	0.06			0.06	0	0
Compound **1** RECOVERED through column chromatography	0.115			0.115	316.29	36.37

Amount aldehydic precursor **6**	0.24 g
Previous waste **1** (Water excluded)	3886.67 g
Previous waste **1** (Water included)	3985.74 g
Previous waste **9** (Water excluded)	1036.13 g
Previous waste **9** (Water included)	1118.66 g
Total waste (Water excluded)	6929.73 g
Total waste (Water included)	7111.33 g
E factor (Water excluded)	28873.89
E factor (Water included)	29630.56
Total cost	259.73 €
Cost for aldehydic precursor **6**	1082.19 €/gr

TTZ5 "SUZUKI-MiYAURA ROUTE" SYNTHESIS

Reagent	Amount (g)	Amount (mL)	Density (g/mL)	Total mass (g)	Price (€/g or €/mL)	Total price (€)
Compound **6**	0.68			0.68	3496.62	2377.70
cyanoacetic acid	0.44			0.44	0.14	0.06
ammonium acetate	0.16			0.16	0.09	0.015
toluene		30	0.867	26.01	0.04	1.074
acetic acid		15	1.05	15.75	0.42	6.33
toluene		150	0.867	130.05	0.04	5.37
HCl(aq.) (0,3M) (2*200 mL)	4.4	400		4.4	0.04	13.84
Water (HCl 0.3 M)		400	1	400	0	0
Na_2SO_4 (drying)	2			2	0.030	0.060
methanol (2x50 mL)		100	0.792	79.2	0.020	2.04
hexane (2x 50 mL)		100	0.659	65.9	0.070	7.02

Amount **TTZ5**	0.69 g
E factor (Water excluded)	93815.76
E factor (Water included)	96384.21
Total cost	2413.52 €
Cost for **TTZ5**	3497.85 €/g

TTZ5 cost "C-H/STILLE ROUTE" SYNTHESIS

Reagent	Amount (g)	Amount (mL)	Density (g/mL)	Total mass (g)	Price (€/g or €/mL)	Total price (€)
Compound **6**	0.68			0.68	800.70	544.48
cyanoacetic acid	0.44			0.44	0.14	0.06
ammonium acetate	0.16			0.16	0.09	0.02
toluene		30	0.867	26.01	0.04	0.93
acetic acid		15	1.05	15.75	0.42	6.64
toluene		150	0.867	130.05	0.04	4.66
HCl(aq.) (0,3M) (2*200 mL)	4.4			4.4	0.03	0.15
Water (HCl 0.3 M)		400	1	400	0	0
Na_2SO_4 (drying)	2			2	0.03	0.06
methanol (2x50 mL)		100	0.792	79.2	0.02	1.61
hexane (2x 50 mL)		100	0.659	65.9	0.07	4.63
Amount **TTZ5**			0.69 g			
Total cost			586.58 €			
Cost for **TTZ5**			850.11 €/g			

TTZ5 cost "ONE-POT C-H ACTIVATION ROUTE" SYNTHESIS

Reagent	Amount (g)	Amount (mL)	Density (g/mL)	Total mass (g)	Price (€/g or €/mL)	Total price (€)
Compound **6**	0.68			0.68	1082.19	735.89
cyanoacetic acid	0.44			0.44	0.14	0.06
ammonium acetate	0.16			0.16	0.09	0.015
toluene		30	0.867	26.01	0.04	1.074

acetic acid		15	1.05	15.75	0.42	6.33
toluene		150	0.867	130.05	0.04	5.37
HCl(aq.) (0,3M) (2*200 mL)	4.4	400		4.4	0.04	13.84
Water (HCl 0.3 M)		400	1	400	0	0
Na_2SO_4 (drying)	2			2	0.030	0.060
methanol (2x50 mL)		100	0.792	79.2	0.020	2.04
hexane (2x 50 mL)		100	0.659	65.9	0.070	7.02
Amount **TTZ5**			0.69 g			
Total cost			771.70 €			
Cost for **TTZ5**			1118.41 €/g			

2.2 Eco-scale rate

Diethyl 2,2-dipentylmalonate (13)

**Parameter**	**Penalty Points**
1-bromopentane (Toxic, Flammable)	10
THF (Highly flammable) (Toxic)	10
Sodium hydride (Flammable)	5
Dropping Funnel	1
Inert atmosphere	1
Cooling to 0 °C	4
Heating > 1h	3
Liquid-liquid extraction	3
Distillation	3
Total Penalty Points	40

2,2-dipentylpropan-1,3-diol (14)

Parameter	Penalty Points
Lithium aluminum hydride (Flammable)	5
Et_2O (Toxic) (Flammable)	10
Dropping funnel	1
Inert atmosphere	1
Cooling to 0 °C	4
Room temperature, 24h	1
Liquid liquid extraction	3
Total Penalty Points	25

3,3-dipentyl-3,4-dihydro-2*H*-thieno[3,4-*b*][1,4]dioxepine (ProDOT, 15)

Parameter	Penalty Points
3,4-dimethoxythiophene (Toxic)	5
Toluene (highly flammable) (Toxic)	10
p-toluenesulfonic acid (Toxic)	5
Soxhlet extractor	1
Inert atmosphere	1
Heating > 1h	3
Liquid liquid extraction	3
Classical chromatography	10
Total Penalty Points	38

3,3-dipentyl-3,4-dihydro-2*H*-thieno[3,4-*b*][1,4]dioxepine-6-carboxaldehyde (16)

Parameter	Penalty Points
THF (Highly flammable) (Toxic)	10

1,6 M solution of *n*-BuLi in hexane (Toxic) (Flammable) (Dangerous for the environment)	15
N,*N*-DMF (Toxic) (Flammable)	10
Glass syringes	1
Inert atmosphere	1
Cooling to < 0 °C	5
Instruments for controlled addition of chemicals	1
Room temperature < 24 h	1
Liquid liquid extraction	3
Classical chromatography	10
Total Penalty Points	57

ProDOT-TzTz (1)

**Parameter**	**Penalty Points**
Dithiooxamide (Toxic)	5
n-butanol (Toxic) (Highly flammable)	10
THF (Highly flammable) (Toxic)	10
chloranil (Toxic) (Dangerous for the environment)	10
Microwave irradiation (Unconventional activation technique)	2
Heating, > 1 h	3
Heating, < 1 h	2
Classical chromatography	10
Total Penalty Points	52

2,5-bis(8-iodo-3,3-dipentyl-3,4-dihydro-2*H*-thieno[3,4-*b*][1,4]dioxepin-6-yl)thiazolo [5,4-*d*]thiazole (2)

Parameter	Penalty Points
• *N*-iodosuccinimide (Toxic)	10
• Chloroform (Toxic)	5
• Ethyl Acetate	10
• Inert gas atmosphere	1
• Room temperature, < 24 h	1
• Total Penalty Points	27

Extended ProDOT-TzTz-aldehyde (8)

Parameter	Penalty Points
pivalic acid (Toxic)	5
potassium carbonate (Toxic)	5
toluene (Highly flammable) (Toxic)	10
Inert atmosphere	1
Degassification	1
Heating, > 1 h	3
Classical chromatography	10
Total Penalty Points	35

1-bromo-4-hexylthiobenzene (18)

Parameter	Penalty Points
1-bromo-4-iodobenzene (**17**) (Toxic)	5
Toluene (highly flammable) (Toxic)	10
Copper iodide (Toxic) (dangerous for environment)	10

1,10-phenanthroline (Toxic + Dangerous for environment)	10
sodium *tert*-butoxide (highly flammable)	5
1-hexanethiol (Toxic) (Highly flammable)	10
ethyl acetate (Toxic) (highly flammable)	10
Inert atmosphere	1
Degassification	1
Heated to reflux > 1h	3
Classical chromatography	10
Total Penalty Points	75

4-(Hexylthio)-*N*-(4-(hexylthio)phenyl)-*N*-phenylaniline (19)

**Parameter**	**Penalty Points**
Toluene (highly flammable) (Toxic)	10
$Pd_2(dba)_3.CHCl_3$ (Toxic)	5
aniline (toxic + dangerous for environment)	10
sodium *tert*-butoxide (highly flammable)	5
Et_2O (Toxic) (Flammable)	10
Inert atmosphere	1
Degassification	1
Heating, > 1 h	3
Liquid-liquid extraction	3
Classical chromatography	10
Total Penalty Points	58

4-Bromo-*N*,*N*-bis(4-(hexylthio)phenyl)aniline (9)

Parameter	Penalty Points
dry $CHCl_3$ (Toxic)	5
N-bromosuccinimide (Toxic)	5
Inert atmosphere	1
Room temperature, < 24 h	1
Liquid liquid extraction	3
Total Penalty Points	15

4-(Hexylthio)-*N*-(4-(hexylthio)phenyl)-*N*-(4-(4,4,5,5-tetramethyl-1,3,2-dioxaborolan-2-yl)phenyl)aniline (3)

Parameter	Penalty Points
Pd(dppf)Cl_2	5
N,N-dimethylformamide (Toxic) + (Flammable)	10
Inert atmosphere	1
Heating, > 1 h	3
Liquid liquid extraction	3
Classical chromatography	10
Total Penalty Points	32

4-(Hexylthio)-*N*-(4-(hexylthio)phenyl)-*N*-(4-(8-(5-(8-iodo-3,3-dipentyl-3,4-dihydro-2*H*-thieno[3,4-*b*][1,4]dioxepin-6-yl)thiazolo[5,4-*d*]thiazol-2-yl)-3,3-dipentyl-3,4-dihydro-2*H*-thieno[3,4-*b*][1,4]dioxepin-6-yl)phenyl)aniline (4)

Parameter	Penalty Points
Pd(dppf)Cl_2	5
Potassium Fluoride (Toxic)	5

Toluene (Toxic) + (Flammable)	10
Inert Gas Atmosphere	1
Degassification	1
Heating > 1 h	3
Classical chromatography	10
Total Penalty Points	35

4-Tributylstannyl-*N*,*N*-(4-hexylthiophenyl)aniline (10)

Parameter	Penalty Points
THF (Highly flammable) (Toxic)	10
1.6 M solution of *n*-BuLi in hexane (Toxic) (Flammable) (Dangerous for the environment)	15
Bu_3SnCl (Toxic + dangerous for environment)	10
ethyl acetate (Toxic + highly flammable)	10
Glass syringes	1
Inert atmosphere	1
Cooling < 0°C	5
Room temperature, < 24 h	1
Liquid-liquid extraction	3
Total Penalty Points	56

TTZ5 precursor aldehyde (6) "SUZUKI-MIYAURA ROUTE" SYNTHESIS

Parameter	Penalty Points
5-formyl-2-thiopheneboronic acid	5
$Pd(dppf)Cl_2$	5
Potassium Fluoride (Toxic)	5

Toluene (Toxic and Flammable)	10
Microwave Irradiation	2
Degassification	1
Heating < 1 h	2
Liquid liquid extraction	3
Classical chromatography	10
Total Penalty Points	43

TTZ5 precursor aldehyde (6) PART A "C-H/STILLE ROUTE" SYNTHESIS

Parameter	Penalty Points
chloroform (Toxic)	5
NBS (Toxic)	5
Inert atmosphere	1
Cooling at –15 °C	5
Liquid liquid extraction	3
Total Penalty Points	19

TTZ5 precursor aldehyde (6) PART B "C-H/STILLE ROUTE" SYNTHESIS

Parameter	Penalty Points
Toluene (highly flammable) (Toxic)	10
Inert atmosphere	1
Degassification	1
Heating, > 1 h	3
Classical chromatography	10
Total Penalty Points	25

TTZ5 precursor aldehyde (6) "ONE-POT C-H ACTIVATION ROUTE" SYNTHESIS

Parameter	Penalty Points
potassium carbonate (Toxic)	5
anhydrous toluene (Toxic) + (Highly Flammable)	10
pivalic acid (Toxic)	5
Inert gas atmosphere	1
Heating, > 1 h	3
Heating, > 1 h	3
Degassification	1
Filtration	1
Classical chromatography	10
Total Penalty Points	39

TTZ5 "C-H/STILLE ROUTE" SYNTHESIS

Parameter	Penalty Points
cyanoacetic acid (Toxic)	5
Toluene (Highly flammable) (Toxic)	10
Inert atmosphere	1
Heating, > 1 h	3
Liquid-liquid extraction	3
Crystallization and filtration	1
Total Penalty Points	23

TTZ5 “ONE-POT C-H ACTIVATION ROUTE” SYNTHESIS

Parameter	Penalty Points
cyanoacetic acid (Toxic)	5
toluene (highly flammable) (Toxic)	10
Inert gas atmosphere	1
Heating, > 1 h	3
Liquid-liquid extraction	3
Crystallization and filtration	1
Total Penalty Points	23

ECO SCALE FINAL CALCULATION

PARAMETER	SUZUKI-MIYAURA ROUTE	C-H/STILLE ROUTE	ONE-POT C-H ACTIVATION ROUTE
Overall yield Penalty Points	49.59438	47.550	48.13
Price for 10 mmol of **TTZ5** Penalty Points (Very expensive > $ 50)	5	5	5
Penalty points of the previous steps	497	495	399
Penalty points **TTZ5** (yield excluded)	23	23	23
Total Penalty Points	574.59	570.55	475.13
Total Eco scale (100- Total Penalty Points)	-474.59	-470.55	-375.13

3 Life Cycle Assessment

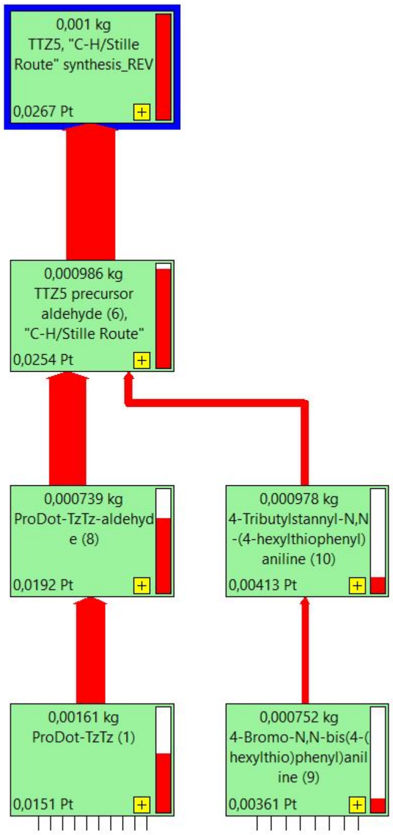

Supplementary Figure 1. ILCD Single Score C-H/Stille Route – Process Flow

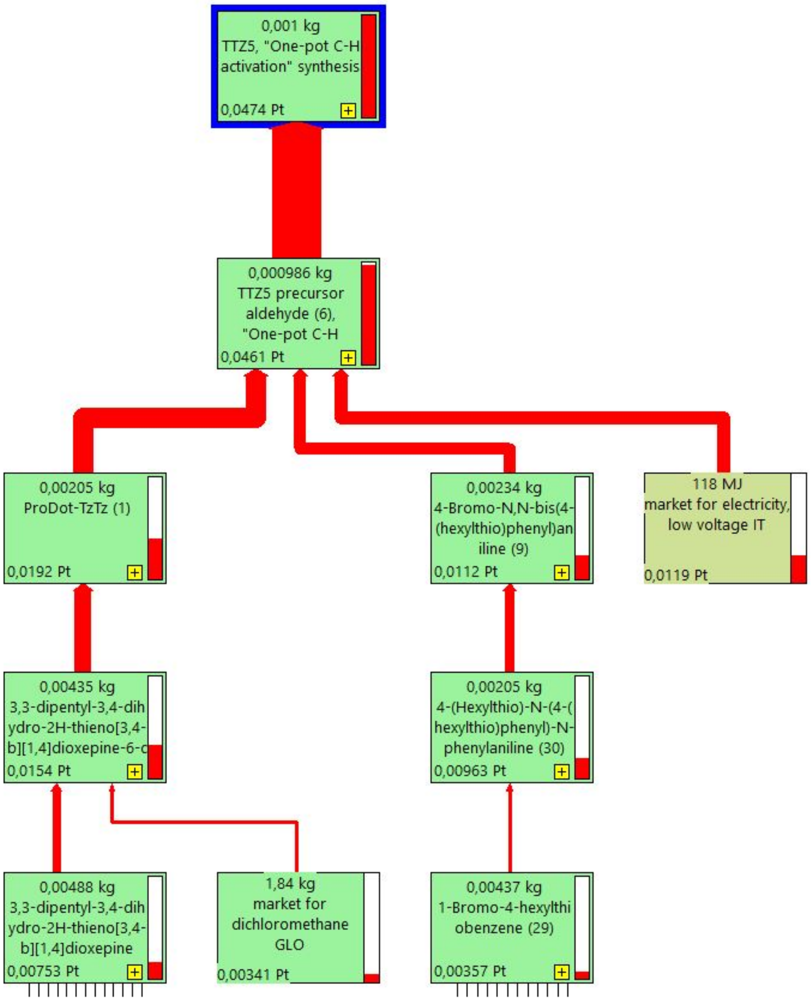

Supplementary Figure 2. ILCD Single Score one-pot C-H Activation Route – Process Flow

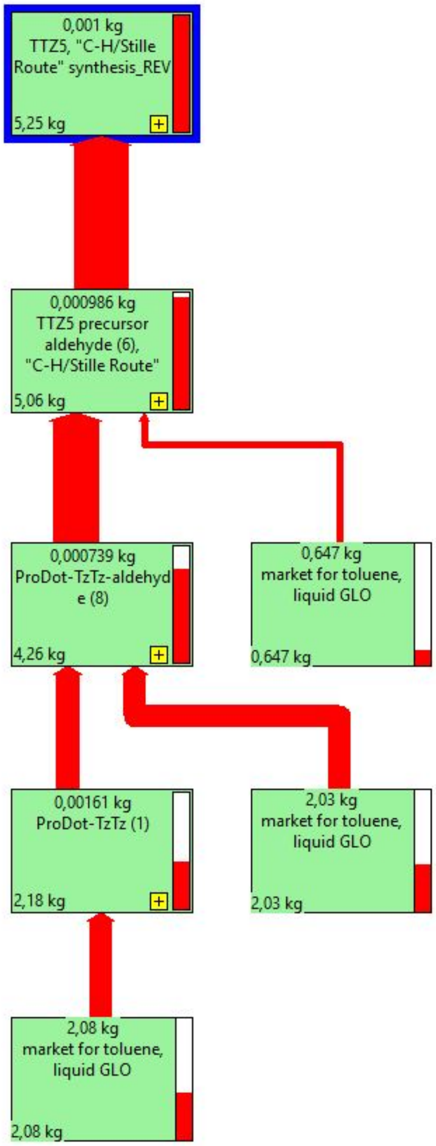

Supplementary Figure 3. ILCD Single Score C-H/Stille Route – Single product Flow (Toluene consumption)

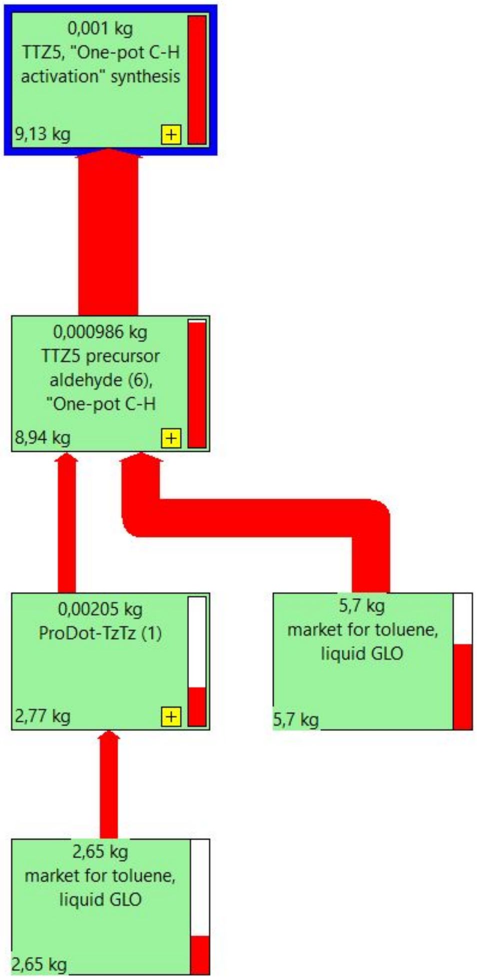

Supplementary Figure 4. ILCD Single Score one-pot C-H Activation Route – Single product Flow Toluene consumption)

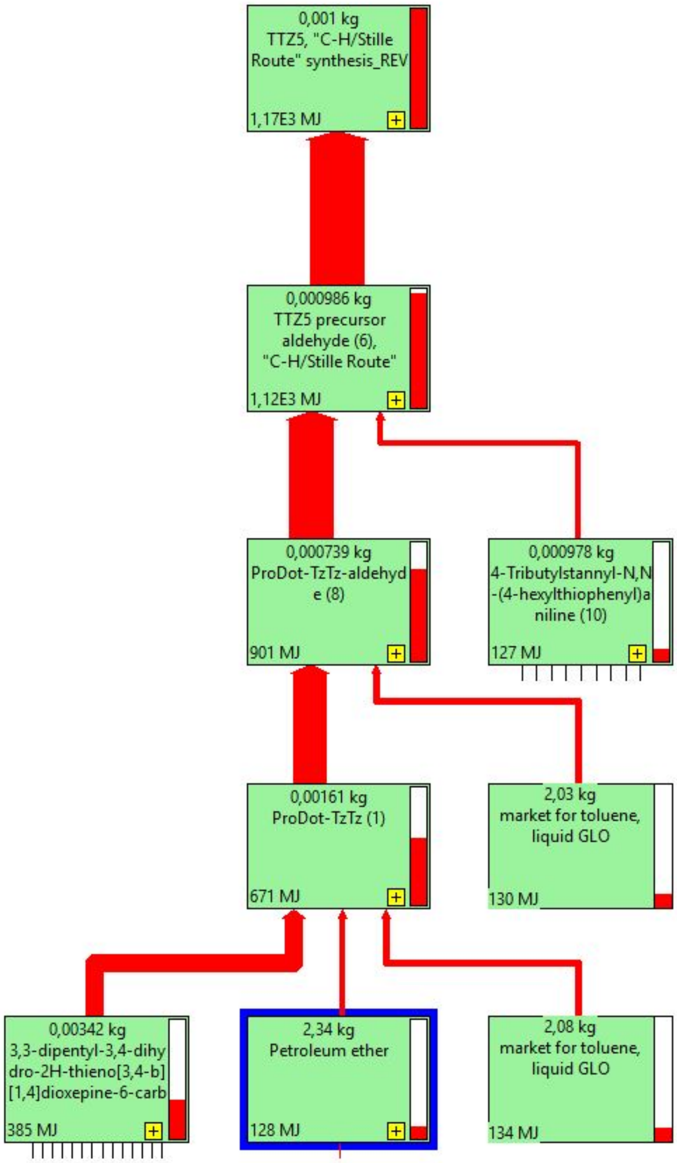

Supplementary Figure 5. CED single score C-H Stille Route – Process Flow

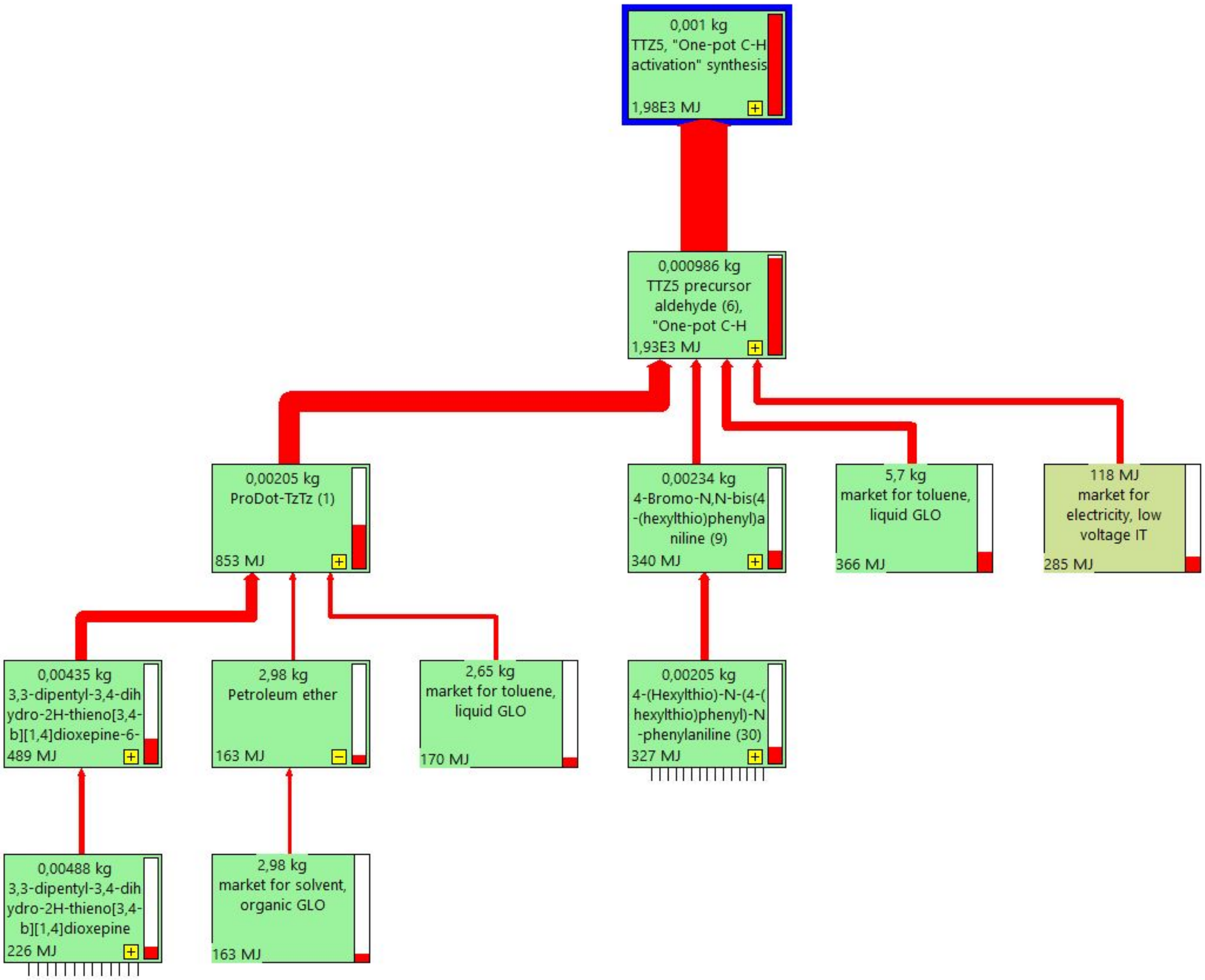

Supplementary Figure 6. CED single score one-pot C-H Activation Route – Process Flow

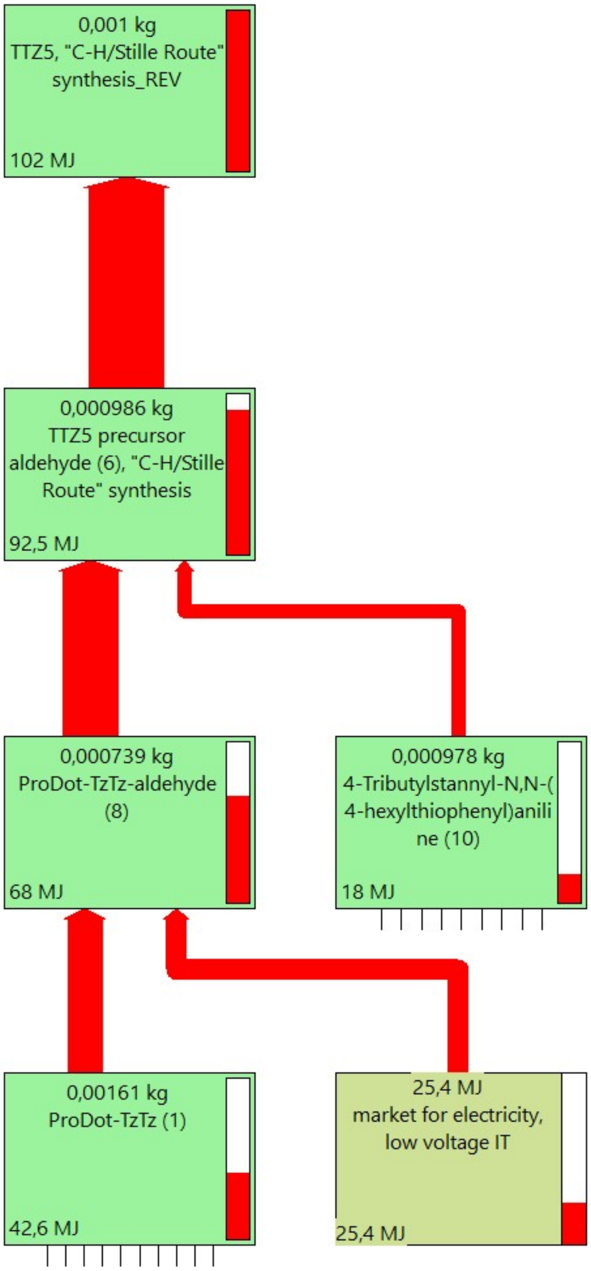

Supplementary Figure 7. CED single score C-H/Stille Route – Single product Flow (direct electricity consumption)

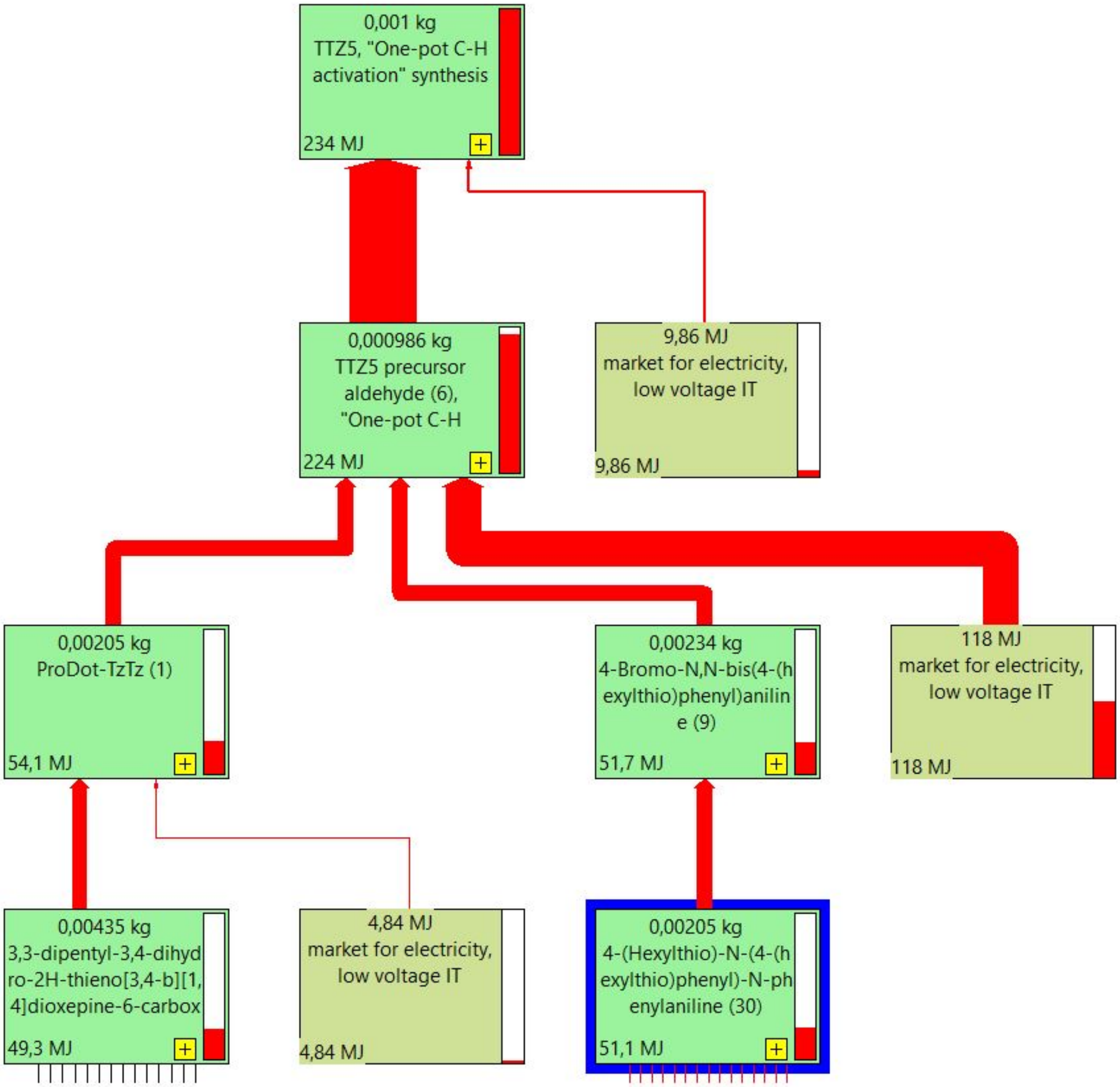

Supplementary Figure 8. CED single score one-pot C-H Activation Route – Single product Flow (direct electricity consumption)
